# MIR4435-2HG: a key player in the novel lncRNA prognostic signatures causes early metastasis after tumor resection and poor prognosis for esophageal squamous cell carcinoma

**DOI:** 10.1186/s12885-025-15299-y

**Published:** 2025-11-24

**Authors:** Pengju Qi, Shuhua Huo, Wenchao Wu, Tengfei Zhang, Wenqian Yang, Jiaping Tang, Yuzhen Liu, Baosheng Zhao

**Affiliations:** 1 Department of Thoracic Surgery, The First Affiliated Hospital of Henan Medical University, Weihui, Xinxiang, Henan Province 453100 China; 2 Esophageal Cancer Institute of Henan Medical University, Weihui, Xinxiang, Henan Province 453100 China; 3 Life Science Research Center, The First Affiliated Hospital of Henan Medical University, Weihui, Xinxiang, Henan Province 453100 China

**Keywords:** ESCC, MIR4435-2HG, Metastasis, PI3K-Akt signaling pathway, Biomarker

## Abstract

**Background:**

Esophageal squamous cell carcinoma (ESCC) is a major histopathological type of esophageal cancer. Tumor metastasis is a critical factor that dramatically worsens the prognosis, leading to a decline in survival rates. Understanding the molecular mechanisms underlying metastasis and identifying reliable biomarkers to predict metastasis susceptibility are essential for improving patient outcomes. This study aims to explore the role of lncRNA MIR4435-2HG in ESCC metastasis susceptibility and its potential as a prognostic biomarker.

**Methods:**

We conducted a genome-wide analysis of gene expression in ESCC tissues from four patients with similar clinical characteristics but divergent prognoses to identify differentially expressed lncRNAs and mRNAs. We focused on the lncRNA MIR4435-2HG, examining its expression levels in correlation with clinical outcomes and its role in the PI3K-Akt pathway through in vitro experiments and bioinformatics analysis.

**Results:**

Differential expression analysis identified 2000 prognosis-related lncRNAs, with MIR4435-2HG showing significant upregulation in tumor tissues of patients with poor prognosis. High expression levels of MIR4435-2HG were associated with advanced tumor stage and short-term survival. The key lncRNA-miRNA-mRNA axes in the prognostic sub-network offer insights into the interactions most relevant to cancer prognosis. The results of in vitro experiments and enrichment analysis suggested that MIR4435-2HG promotes tumor proliferation and metastasis by activating the PI3K-Akt pathway.

**Conclusion:**

MIR4435-2HG may represent a potential biomarker associated with metastasis and poor prognosis. Its possible involvement in the PI3K-Akt pathway warrants further validation and investigation in larger clinical cohorts.

**Supplementary Information:**

The online version contains supplementary material available at 10.1186/s12885-025-15299-y.

## Introduction

The latest global cancer statistics in 2020 show that esophageal cancer (ESCA) ranked seventh in incidence and sixth in mortality among malignant tumors [1]. According to histopathological types, ESCA is mainly divided into squamous cell carcinoma (ESCC) and adenocarcinoma (EAC), with the incidence of the former being more than 90% in China [2]. Tumor metastasis is the main driver of poor prognosis in ESCC patients [3]. Therefore, identifying metastasis-related biomarkers and implementing biological interventions are critical for improving ESCC patient outcomes [4].

LncRNA has emerged as a crucial regulator in tumorigenesis and progression through comprehensive research [5]. It plays a significant role in epigenetic and expression regulation at both transcriptional and post-transcriptional levels, as well as in control of chromosome stability [6]. Consequently, the exploration of lncRNA’s involvement in the pathological mechanism and its potential application as biomarkers or therapeutic targets for tumor diagnosis and prognosis has attracted widespread attention. Compared to mRNA, lncRNA has higher stability due to abundant secondary structures and protection of exosomes. Furthermore, the detection of numerous lncRNAs in human blood and urine provides great convenience for non-invasive diagnosis [7–9].

In clinical practice, the prognosis of ESCC patients is primarily evaluated using the traditional Tumor Node Metastasis (TNM) staging system [10]. However, our team has observed a perplexing phenomenon during long-term surgical practice: a subset of ESCC patients with identical pathological characteristics, tumor stage, and surgical factors exhibit varying prognostic outcomes. Some ESCC patients with early-stage experienced lymphatic metastasis as early as one to six months after surgery, while others with advanced-stage did not experience metastasis even after five or more years post-surgery. This clinical observation suggests that individual differences in tumor metastasis susceptibility exist among ESCC patients. We term these differences “metastasis susceptibility”, which might be mediated by specific signaling profiles related to tumor metastasis. Identifying and studying these molecular markers is crucial for enhancing the TNM staging system, enabling more precise diagnosis and targeted treatment [11]. However, there is a lack of large-scale clinical studies investigating ESCC patients with identical TNM stages but differing outcomes, and the specific molecular markers involved remain elusive.

To identify metastasis-susceptibility related lncRNAs (MESUlncs) in ESCC, we conducted RNA-seq analysis of lncRNA and mRNA expression in two pairs of ESCC tumor tissues and adjacent paired tissues. These patients exhibited substantial agreement in clinical information and pathological features but had markedly different prognoses, with the poor-prognosis group showing overall survival times of less than 1 year and the good-prognosis group exceeding 8 years. Using both our sequencing data and publicly available datasets (e.g., TCGA and GEO), this study aimed to screen for candidate lncRNAs associated with metastasis susceptibility and to investigate their clinical relevance and functional mechanisms through integrated bioinformatic and experimental approaches. In doing so, we sought to provide novel insights into the molecular determinants of ESCC metastasis.

## Materials and methods

### Summary of the materials and methods

In the Materials and Methods section, the collection of ESCC samples and the acquisition of the metastasis-susceptibility related genes (MESUGs) were described in detail. Other parts, such as the evaluation and construction of the MESU-related prognostic model, molecular typing, and drug sensitivity analysis, are included in the Supplementary Materials. Detailed information can be found in Additional file 1.

### Patients and ESCC samples collection

Samples for RNA-seq and RT-qPCR analyses were all sourced from our clinical sample database at Department of Thoracic Surgery, The First Affiliated Hospital of Henan Medical University. The study was conducted in accordance with the Declaration of Helsinki and was approved by the Committees for Ethical Review of Research at Henan medical University (no. EC-023–457). All patients who underwent radical resection of esophageal cancer had not received any radiotherapy or chemotherapy prior to the surgery, and postoperative tissues were diagnosed as ESCC through pathological examination. The ESCC tumor and paired-adjacent tissues were stored at −80 ℃. As of December 31, 2022, a total of 1322 tissues had been collected in our clinical sample database, and specialized staff have registered the clinical data of these patients and followed up the prognosis. Following strict accordance to the inclusion criteria (Patients in stage I, II, and III, with significant prognostic differences between January 1, 2014 and December 31, 2017) and exclusion criteria (Patients with incomplete clinical data, postoperative complications, missing samples, or whose death were not caused by tumor), we select 111 patients. This included 46 patients in the poor-prognosis group (OS ≤ 1 year) and 65 patients in the good-prognosis group (OS ≥ 5 years).

### Identification of the MESUGs

RNA-seq [12], conducted by Shanghai Personalbio Technology Co.,Ltd., was employed to find the differential expressed genes (DEGs) in tumor tissues of ESCC patients with different prognosis, while ensuring consistency in pathology type, differentiation grade, neoplasm staging, surgeon and surgical method across all cases. DEGs were screened and obtained using the R package ‘limma’ with *P* < 0.05 and │log2 fold change (FC)│ >1. The same approach was then applied to obtain DEGs in tumors compared to pericarcinous or normal tissues, using both our RNA-seq data and publicly databases (TCGA, UCSC XENA and GEO: GSE53624 and GSE53625). The overlapping genes were identified using a Venn diagram. Genes up-regulated in tumor tissues and highly expressed in poor-prognosis group, as well as those up-regulated in pericarcinous or normal tissues and highly expressed in good-prognosis group, were named MESUGs.

### Acquisition of biomarkers related to early postoperative metastasis and poor prognosis

The biomarker related to early postoperative metastasis and poor prognosis was selected from characteristic genes in the MESU-related prognostic model. The differential expression and disease-free survival curve of MIR4435-2HG in ESCA tumor and pericarcinous tissues were generated using the online analysis site GEPIA (http://gepia.cancer-pku.cn/). The R package ‘ggpubr’ and ‘ComplexHeatmap’ were used for differential analysis and correlation heatmap generation, respectively. Survival analysis for each group was evaluated using Kaplan Meier (KM) curves. Total RNA was extracted from tumors using Trizol reagent (Ambion, Carlsbad, USA), and quantitative Real-time PCR (RT-qPCR) was performed in a 10µL reaction volume using a qPCR detection system (Applied biosystems, Singapore). The primer sequence of MIR4435-2HG is: Forward CACAGAGCTTTCCCTTTATCAG and Reverse CTGATAAAGGGAAAGCTCTGTG.

### Statistical analysis

All statistical analyses were performed using R software (4.3.1). The Chi-square test was employed to compare the clinicopathological features between different groups or clusters. The log-rank test was conducted to analyze the significance of Kaplan-Meier survival differences between groups or clusters. The Wilcoxon test was performed to assess immune cell infiltration levels between risk groups and to check MIR4435-2HG expression levels between different prognostic groups. Expression levels were analyzed using the 2^−ΔΔCT^ method, with each assay repeated three times. A statistically significant difference was set at *P* < 0.05.

## Results

### Acquisition of the differentially expressed long non-coding RNAs (DElncs)

**Fig. 1:**
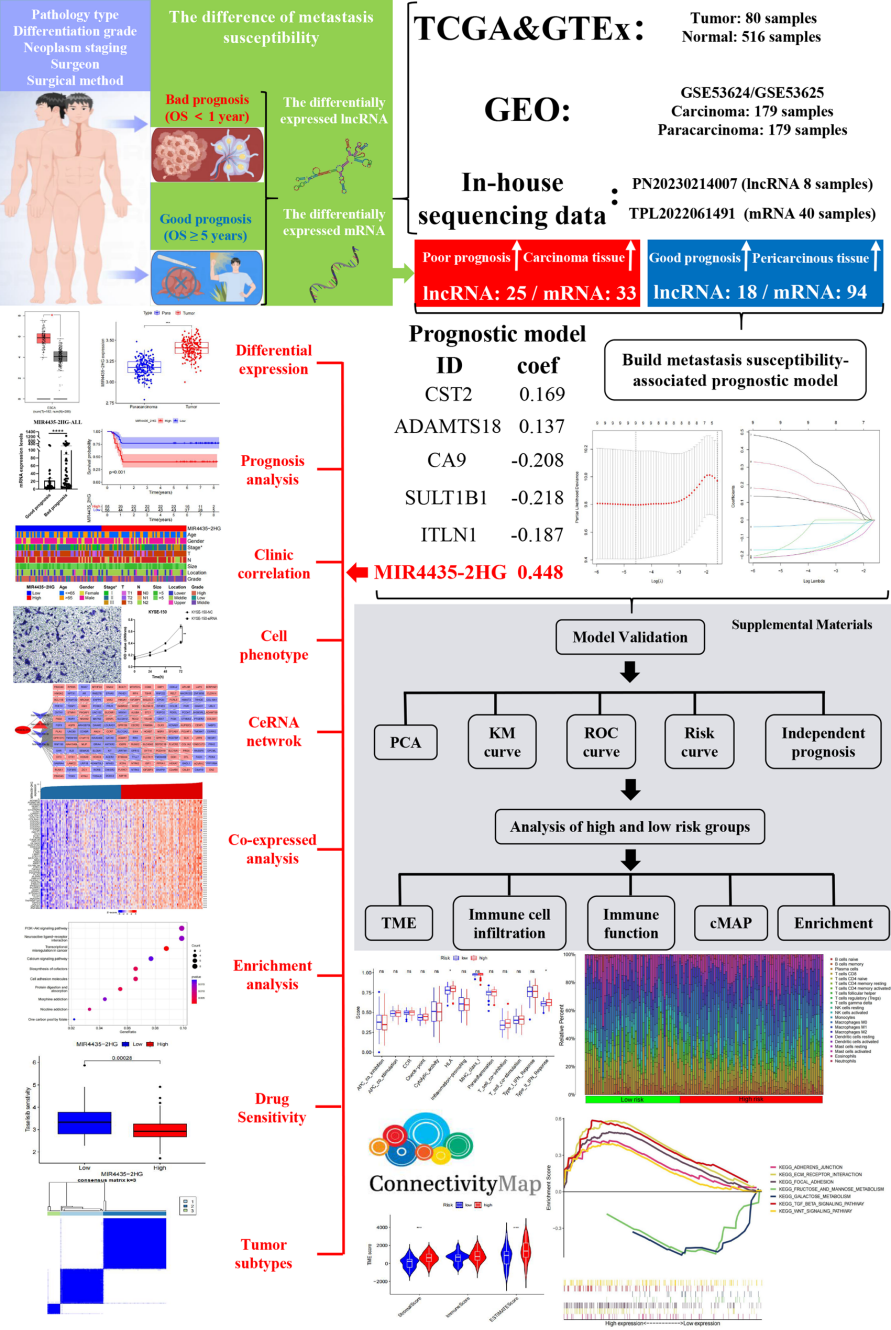
Flowchart of this study

A flowchart of the technical strategy used in this study is presented in Fig. 1. Initially, we searched our clinical sample database (1,322 esophagectomy cases with available follow-up data) and screened four patients with nearly identical pathological and clinical features. These patients were all male, over 60 years old, and shared the same stage, tumor(T), nodal status(N), tumor site, tumor type, and anastomosis method. To minimize the influence of differentiation degree, both high- and low-differentiated tumors were included in the good- and poor-prognosis groups, thereby balancing this factor across the cohort. Although the surgery dates differed, all procedures were performed by the same surgeon using the same surgical approach, ensuring technical consistency. Nevertheless, the prognosis of patients 723 and 1033 was notably poor, with tumor metastasis occurring less than one year after surgery, ultimately resulting in death. In contrast, patients 386 and 433 had excellent prognosis, with no tumor metastasis even after 8 years. Therefore, patients 723 and 1033 were classified into poor-prognosis group, while patients 386 and 433 were categorized into good-prognosis group (Table 1).

**Table 1:** Patient information for lncRNA-seq

ID	Gender	Age	Stage	T	*N*	Tumor size(cm)	Tumor location	Tumor type	Differentiation degree	Anastomoses methods	Surgery Date	Overall survival(mouth)	Overall survival(year)
723	Male	61	ⅡA	T3	N0	5.3	lower esophagus	ulcerative type	high	esophagogastrostomy below aortic arch(surgicalstapler)	2016.02.23	2	
1033	Male	60	ⅡA	T3	N0	4.7	lower esophagus	ulcerative type	low	esophagogastrostomy below aortic arch(surgicalstapler)	2018.06.21	9	
386	Male	68	ⅡA	T3	N0	3.1	lower esophagus	ulcerative type	low	esophagogastrostomy below aortic arch(surgicalstapler)	2014.01.23		> 8
433	Male	71	ⅡA	T3	N0	4.8	lower esophagus	ulcerative type	high	esophagogastrostomy below aortic arch(surgicalstapler)	2014.04.18		> 8

**Fig. 2:**
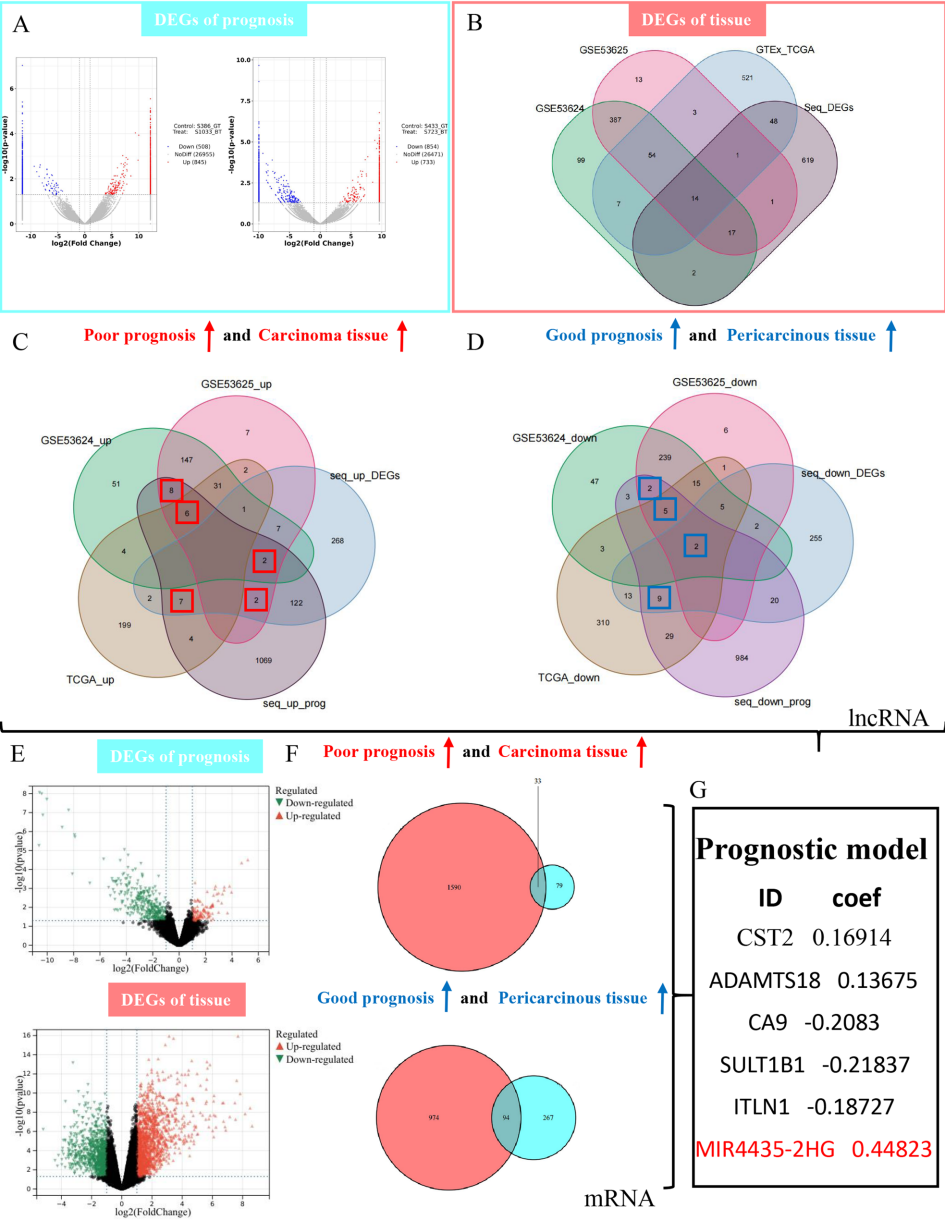
Identification of MESUGs and screening of target genes. **A** Volcano plots depicting DElncs between patients with poor prognosis (patients 723 and 1033) and those with good prognosis (patients 386 and 433). **B** VENN diagram showing overlapping DElncs between tumor and pericarcinous tissues across internal and external datasets (TCGA&GTEx, GSE53624, and GSE53625). **C** Selection of risky lncRNAs that were up-regulated in tumor tissues and highly expressed in the poor prognosis group. The genes that were up-regulated in tumor tissues in at least two data sets were marked with red boxes. **D** Selection of protective lncRNAs that were up-regulated in pericarcinous tissues and highly expressed in the good prognosis group. The genes that were up-regulated in pericarcinous tissues in at least two data sets were marked with blue boxes. **E** Volcano plots of prognosis-related DEmRNAs and DEmRNAs from tumor and pericarcinous tissues in 20 ESCC patients with significantly different prognosis. **F** VENN diagrams showing the identification of risky and protective mRNAs. A total of 43 DElncs and 127 DEmRNAs constitute the gene-set of MESUGs. **G** LASSO regression model highlighting MIR4435-2HG as the most significant gene with the highest effect value of 0.44823

We compared the sequencing data of tumor tissues from patients with the same degree of differentiation, with patients 386 and 433 from good-prognosis group serving as controls (Fig. 2A). Through Venn merging, we identified a total of 2000 differentially expressed lncRNAs (DElncs) that were defined as prognosis-related DElncs, of which 274 were overlapping genes. Next, we analyzed the sequencing data of pericarcinous and tumor tissues from these four patients, with all pericarcinous tissues serving as the control group. This analysis yielded a total of 702 DElncs. In order to avoid false positive or false negative results due to the small sample size, we combined data from three external datasets (TCGA&GTEx, GSE53625, and GSE53624) to acquire DElncs in pericarcinous and tumor tissues. This approach aimed to minimize the loss of potential biomarkers to the greatest extent possible (Fig. 2B).

### Identification of the geneset of the metastasis-susceptibility related genes (MESUGs)

We filtered DElncs that exhibited up-regulated in tumor tissues across at least two cohorts and were highly expressed in poor-prognosis group, designating them as risk lncRNAs (Fig. 2C, the 25 DElncs in red boxes). This dual criterion ensures that the selected genes are robust indicators of aggressive tumor behavior and poor clinical outcomes. Accordingly, protective lncRNAs, which were up-regulated in pericarcinous tissues and exhibited high expression levels in good-prognosis group, were identified using a Venn diagram (Fig. 2D, the 18 DElncs in blue boxes).

For mRNA analysis, pericarcinous and tumor tissues of 20 ESCC patients with significant prognostic differences were subjected to mRNA-seq (Fig. 2E). Risk mRNAs (Fig. 2F, the 33 overlapping mRNAs) and protective mRNAs (Fig. 2F, the 94 overlapping mRNAs) were obtained using the same filtering method as for lncRNA. Considering that the sample size of mRNA-seq was sufficient to accurately obtain the differentially expressed mRNAs (DEmRNAs), we did not merge external databases, which also reduced the analytical redundancy. Ultimately, these 43 DElncs and 127 DEmRNAs made up the geneset of the MESUGs, providing a new basis for ESCC metastasis treatment.

### Acquisition of characteristic gene MIR4435-2HG

LASSO regression was employed to identify characteristic genes with the greatest predictive ability for the target variable that was early metastasis after tumor resection and poor prognosis in this study. Consequently, we used the geneset of MESUGs to build a prognostic model in the GSE53625 dataset, containing the largest number of patients. After evaluation, it was found that this prognostic model accurately predicted the overall survival and recurrence risk of ESCC patients by quantifying risk scores (Fig. S1), and the construction of the nomogram further enhanced its clinical applicability (Fig. S1-g and S2). Our analysis revealed that the lncRNA MIR4435-2HG exhibited the highest effect value, at 0.44832 (Fig. 2G).

In high-risk patients, higher proportions of M0 macrophages and resting mast cells, along with fewer plasma cells, suggested a weaker antitumor immune response, contributing to poorer prognosis (Fig. S3A, B). Despite this, these patients showed increased HLA and Type_II_IFN Response activities, indicating a compensatory mechanism where remaining immune cells were more activated or immune pathways were upregulated to counteract the tumor’s aggressiveness (Fig. S3C). The GSEA results indicated that high-risk tumors showed significant enrichment in pathways related to cell adhesion, migration, and cytoskeletal regulation, which facilitated metastasis and invasion, explaining the poorer outcomes in these patients (Fig. S3D). Potential therapeutic drugs, including huperzine-a and 3,3’-diindolylmethane, were screened to counteract gene expression changes in high-risk patients (Table S1).

### MIR4435-2HG as a biomarker for early postoperative metastasis and poor prognosis

**Fig. 3:**
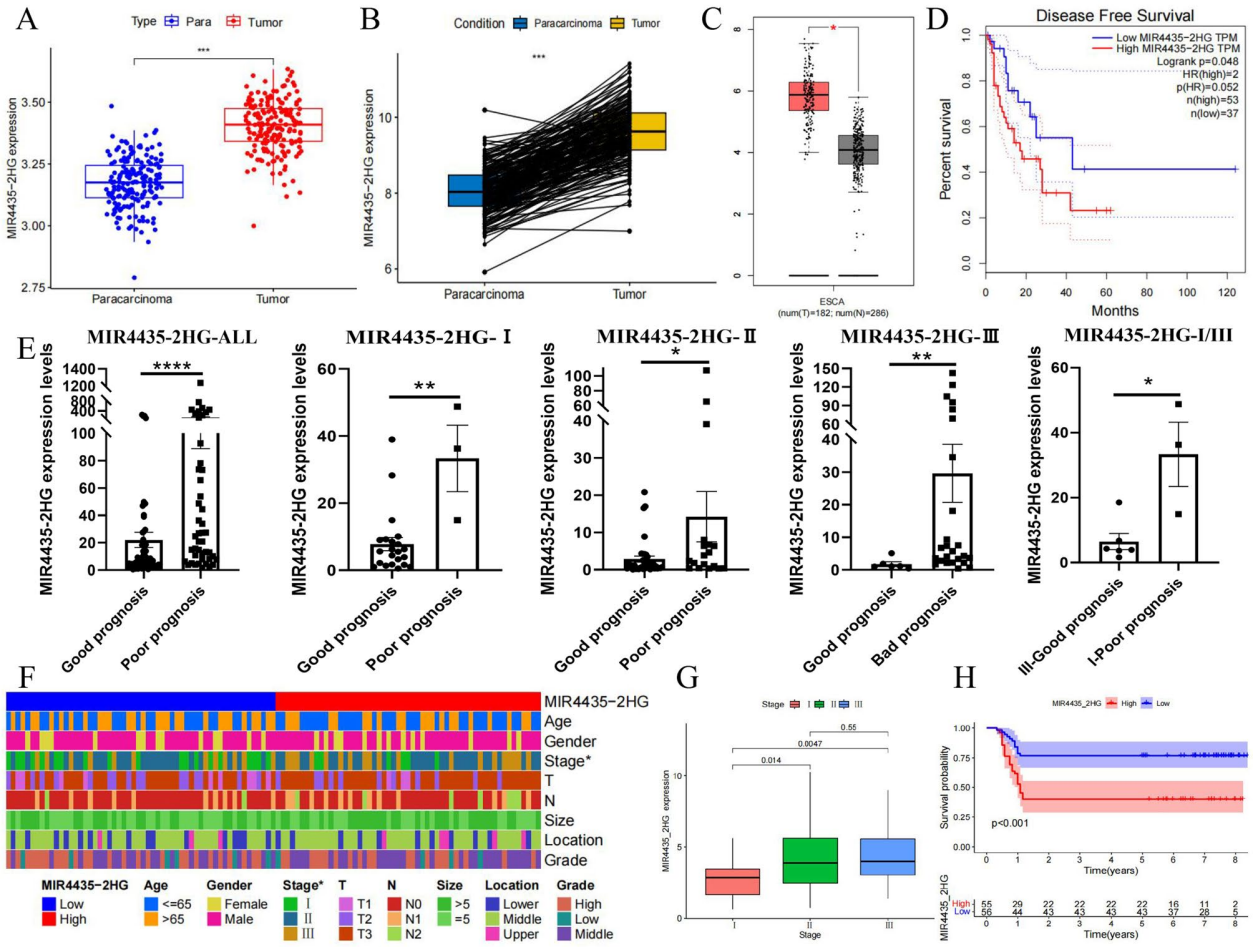
Analysis of expression pattern and clinical correlation of MIR4435-2HG. **A**-**B** Analysis of expression pattern of MIR4435-2HG in tumor and pericarcinous tissues in GSE53625 dataset. **C** MIR4435-2HG expression in ESCA patients showing significant up-regulation in tumor tissues. **D** DFS in TCGA-ESCA (GEPIA). Patients were ranked by MIR4435-2HG expression; high = top 30% (*n* = 53), low = bottom 20% (*n* = 37), middle 50% excluded. High expression was associated with shorter DFS (log-rank test; *P* = 0.048). **E** Expression analysis of MIR4435-2HG in tumor tissues from 111 ESCC patients. In patients with the same stage, those with poor prognosis have higher MIR4435-2HG expression. Even in stage I patients, higher MIR4435-2HG expression often correlates with worse prognosis. The grouping was based on prognostic status (OS ≤ 1 year vs. OS ≥ 5 years), rather than the median expression value. **F** Heatmap illustrating the correlation between MIR4435-2HG expression and various clinicopathological parameters (Stage: *p* = 0.0124). **G** Correlation analysis between MIR4435-2HG expression and Stage. **H** KM survival curves compare overall survival between high and low MIR4435-2HG expression groups

Following RNA-seq analysis, MIR4435-2HG was highly expressed in tumor tissues than pericarcinous tissues across multiple datasets, including our RNA-seq data, GSE53624 and GSE53625 datasets (Fig. 3A, B). Moreover, utilizing an online analysis website ‘GEPIA’, we conducted a differential expression analysis of MIR4435-2HG in ESCA patients and confirmed a substantial increase in its expression within tumor tissues (Fig. 3C). Concurrently, ESCA patients with high expression of MIR4435-2HG showed a higher risk of tumor recurrence (Fig. 3D, log-rank *p* = 0.048). In order to further study the relationship between MIR4435-2HG expression and its implication for early postoperative metastasis and poor prognosis in ESCC patients, we collected 111 tumor tissues from ESCC patients undergoing radical resection of esophageal cancer. As anticipated, MIR4435-2HG was highly expressed in poor-prognosis group, even among patients at the same stage (Fig. 3E, ALL: *p* < 0.0001; stage I: *p* = 0.0061; Stage II: *p* = 0.0489; Stage III: *p* = 0.0061; Stage I/III: *p* = 0.0238.). We were also concerned about patients with poor prognosis at stage I and good prognosis at stage III. Patients with high expression levels of MIR4435-2HG, even if they were at stage I, were also prone to early postoperative metastasis, resulting in poor prognosis (Fig. 3E). The heatmap in Fig. 3F demonstrates a comprehensive overview of the correlation between MIR4435-2HG expression levels and various clinicopathological parameters. The high expression group of MIR4435-2HG displayed a significantly positive correlation with the stage, indicating that this gene may play a role not only in early postoperative metastasis but also in progression and severity of ESCC (Table S2, Fig. 3F, G). Patients exhibiting high MIR4435-2HG expression demonstrated significantly poorer overall survival compared to those with low expression (*P* < 0.001), underscoring the potential of MIR4435-2HG as a prognostic biomarker (Fig. 3H).

### Molecular typing based on MIR4435-2HG and CASC15

**Fig. 4:**
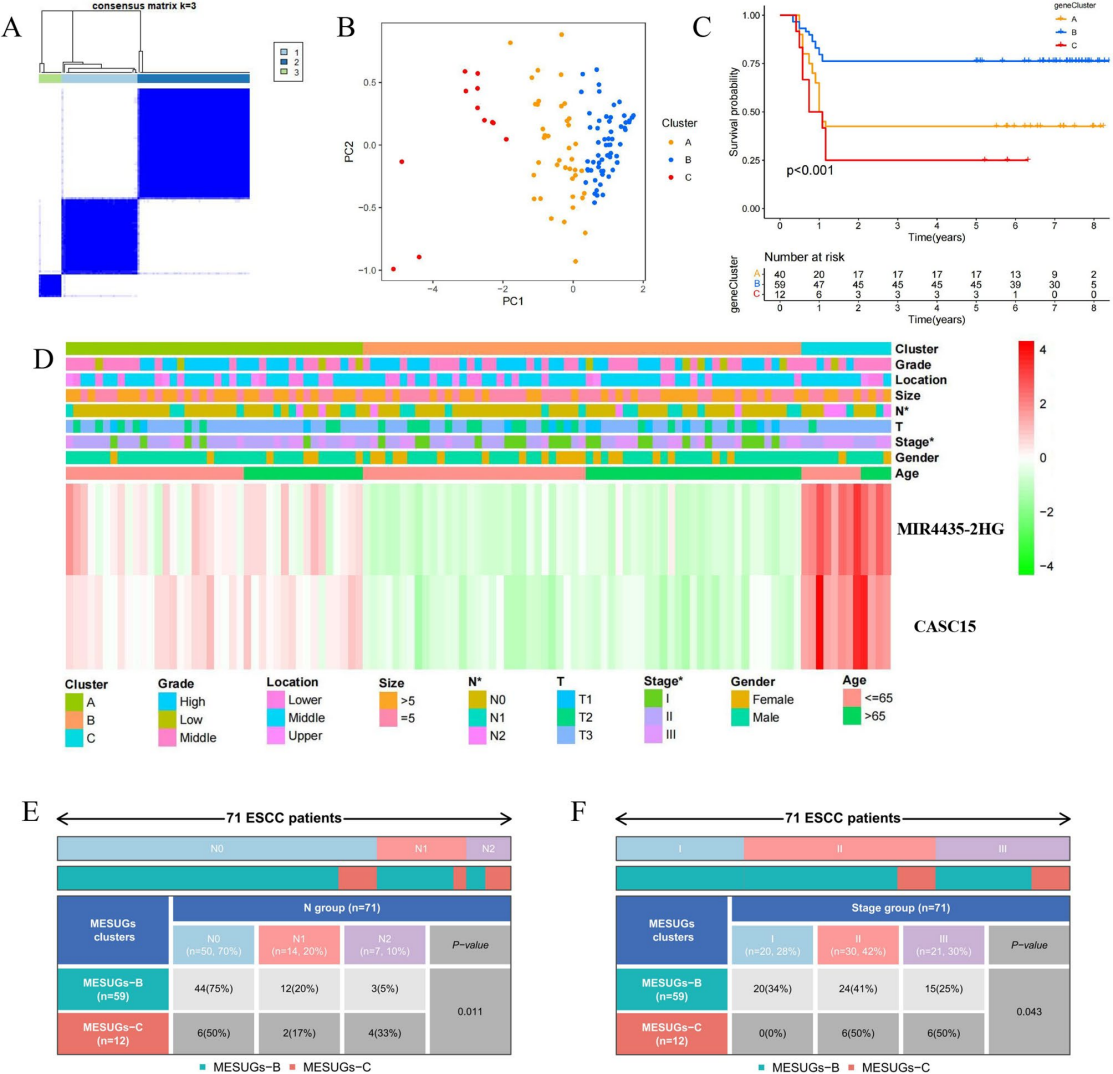
Molecular typing based on MIR4435-2HG and CASC15. **A** Consensus clustering matrix for k = 3; ESCC patients are divided into three clusters. **B** PCA plot confirming the separation of patients into three distinct clusters. **C** KM survival curves showing the survival advantage of cluster **B** over clusters **A** and **C**. **D** Heatmap illustrating the correlation between different clusters and various clinicopathological parameters. **E**-**F** The distribution characteristic of the pathological stage-N and stage in the cluster **B** and **C**

To achieve the goal of precision therapy, we explored molecular typing in ESCC patients. However, the expression level of MIR4435-2HG alone was insufficient for typing. Therefore, we incorporated an additional biomarker, CASC15, which was the only gene among the MESUGs that could generate a significantly different KM curve in the external data set (GSE53625) and had been experimentally validated. Three different clusters were determined by the unsupervised clustering method as foundations for more accurate treatment strategies (Fig. 4A). All patients were clearly divided into three sections according to PCA, further confirming the presence of three remarkably different subtypes (Fig. 4B). KM curves for these three clusters revealed the prominent survival advantage in cluster B (Fig. 4C). Furthermore, the expression levels of MIR4435-2HG and CASC15 were markedly higher in cluster C, which associated with higher grade and advanced disease stages (Fig. 4D). The specific distribution of these two characteristics in clusters B and C was illustrated in Figs. 4E, F, revealing a significantly higher proportion N2 or Stage III patients in cluster C compared to cluster B. Compared to the clinical association analysis based solely on MIR4435-2HG expression levels, the dual-gene tumor subtyping provided a more robust prediction of adverse clinical events.

### Cell proliferation and migration after MIR4435-2HG knockdown

**Fig. 5:**
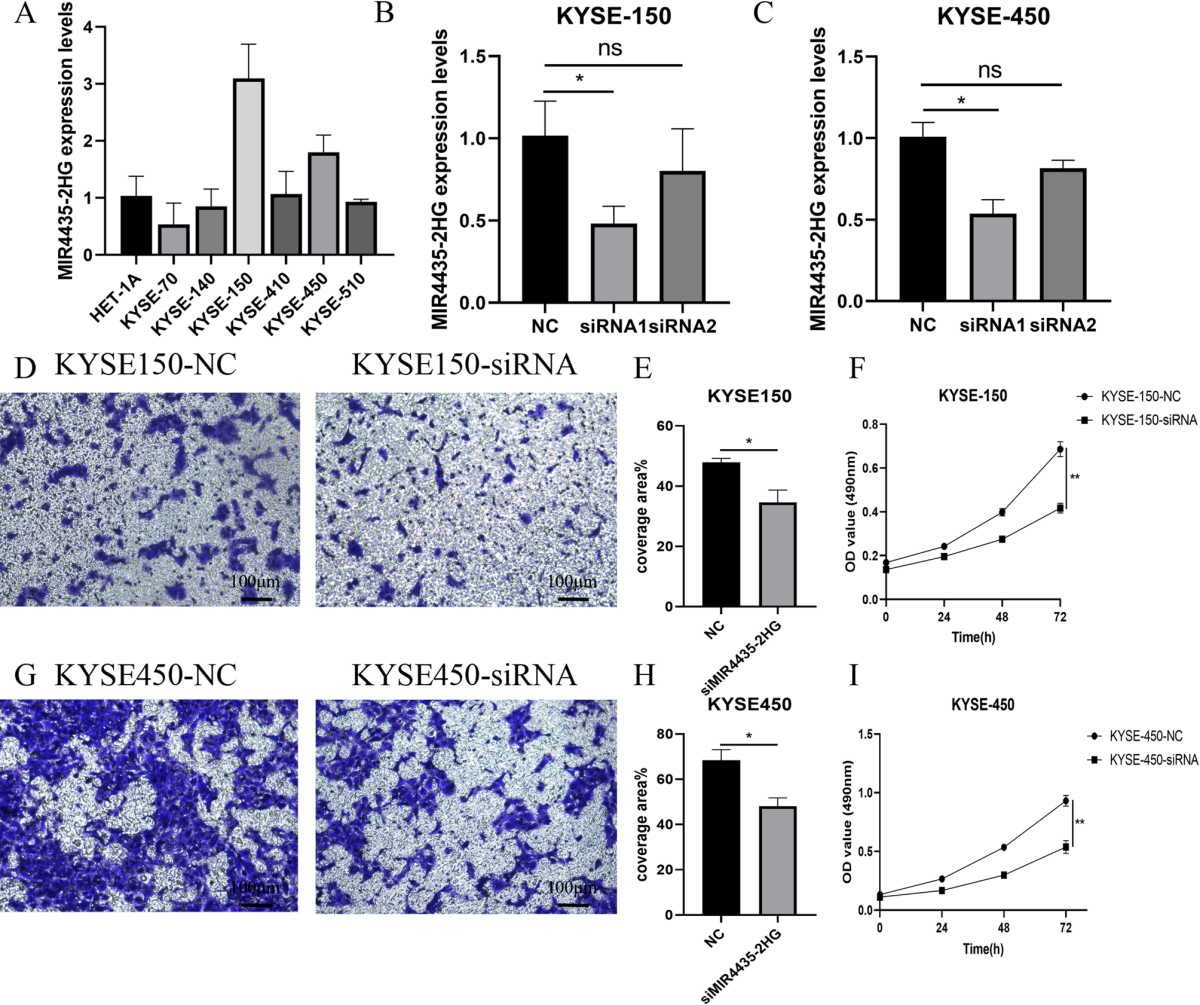
Functional validation of MIR4435-2HG in ESCC Cells. **A** RT-qPCR analysis of MIR4435-2HG expression. **B**-**C** Verification of knockdown efficiency of MIR4435-2HG in KYSE-150 and KYSE-450 cells. **D**-**E**, **G**-**H** Transwell assays showing the inhibitory effect of MIR4435-2HG knockdown on cell migration. **F**, **I** MTT assays indicating that the proliferation ability of ESCC cells decreased after MIR4435-2HG knockdown

Through gene knockdown, we validated the functional role of MIR4435-2HG in ESCC cell behavior to determine its potential as a therapeutic target. The results of RT-qPCR depicted varying the expression levels of MIR4435-2HG across different cell lines, notably higher in KYSE-150 and KYSE-450 cells compared to others (Fig. 5A). We validated the knockdown efficiency of two siRNAs in KYSE150 and KYSE450 cells and found that only siRNA1 significantly reduced gene expression (Fig. 5B, C, KYSE-150: *p* = 0.0172; KYSE-450: *p* = 0.0187.), which was then used for subsequent analyses. MTT and Transwell assays demonstrated that knockdown of MIR4435-2HG significantly inhibited the proliferation (Fig. 5F, I, KYSE-150: *p* = 0.0022; KYSE-450: *p* = 0.0022.) and migration abilities (Fig. 5D, E and G, H, KYSE-150 and KYSE-450: *p* < 0.05.) of different ESCC cells.

### Construction of CeRNA network with MIR4435-2HG as the core

**Fig. 6:**
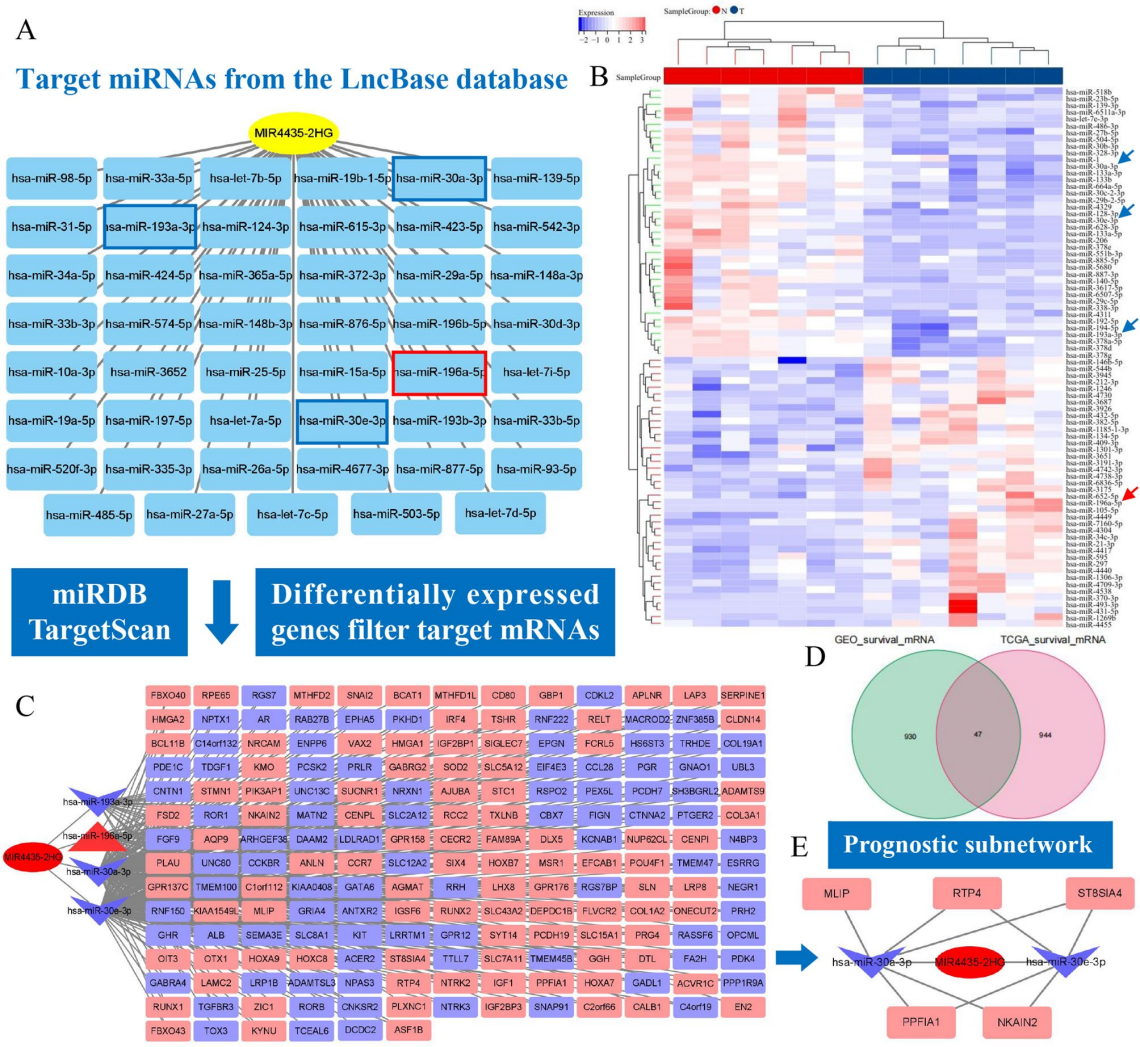
Construction of the ceRNA network and prognostic sub-network. **A** LncBase was used to predict the target miRNA of MIR4435-2HG. The red and blue boxes indicating the DEmis in tumor and pericarcinous tissues. **B** Identification of DEmis in the GSE97051 dataset for optimizing lncRNA-miRNA networks. **C** The construction of lncRNA-miRNA-mRNA network contained 188 DEmRNAs, in which red representing high expression in tumor tissues and blue representing the high expression in pericarcinous tissues. **D** The prognostic sub-network was constructed by identifying prognostic genes from TCGA and GSE53625 data sets. **E** Visualization of the prognostic sub-network, highlighting key lncRNA-miRNA-mRNA axes

To explore the role of MIR4435-2HG in gene expression regulation and its potential biological functions, we constructed a ceRNA network. First, we identified 47 target miRNAs of MIR4435-2HG using the LncBase database (Fig. 6A). Next, from the GSE97051 data set, we obtained 82 DEmis between tumor and pericarcinous tissues (Fig. 6B) and retained those overlapping with target miRNAs to streamline the ceRNA network. Subsequently, we predicted the target mRNAs of the four miRNAs (hsa-miR-30a-3p, hsa-miR-193a-3p, hsa-miR-196a-5p and hsa-miR-30e-3p) using the miRDB and TargetScan databases. We also screened DEmRNAs in ESCC tumor and pericarcinous tissues based on our RNA-seq data, identifying 2691 genes (Fig. 2E). These DEGs were used to filter the target mRNAs, resulting in a ceRNA network comprising 188 differentially expressed target mRNAs (Fig. 6C). Constructing a prognostic sub-network could help in deeply understanding disease mechanisms, discovering new biomarkers and targets, and advancing the development of precision medicine. By mining the results from the ESCC cohort in the TCGA database and the GSE53625 cohort in the GEO database, we identified a total of 1921 prognosis-related genes (Fig. 6D), which were used to construct the prognostic sub-network. The prognostic sub-network in Fig. 6E showed valuable potential lncRNA-miRNA-mRNA axes regulating five risk genes.

### Analysis the potential pathway of MIR4435-2HG

**Fig. 7:**
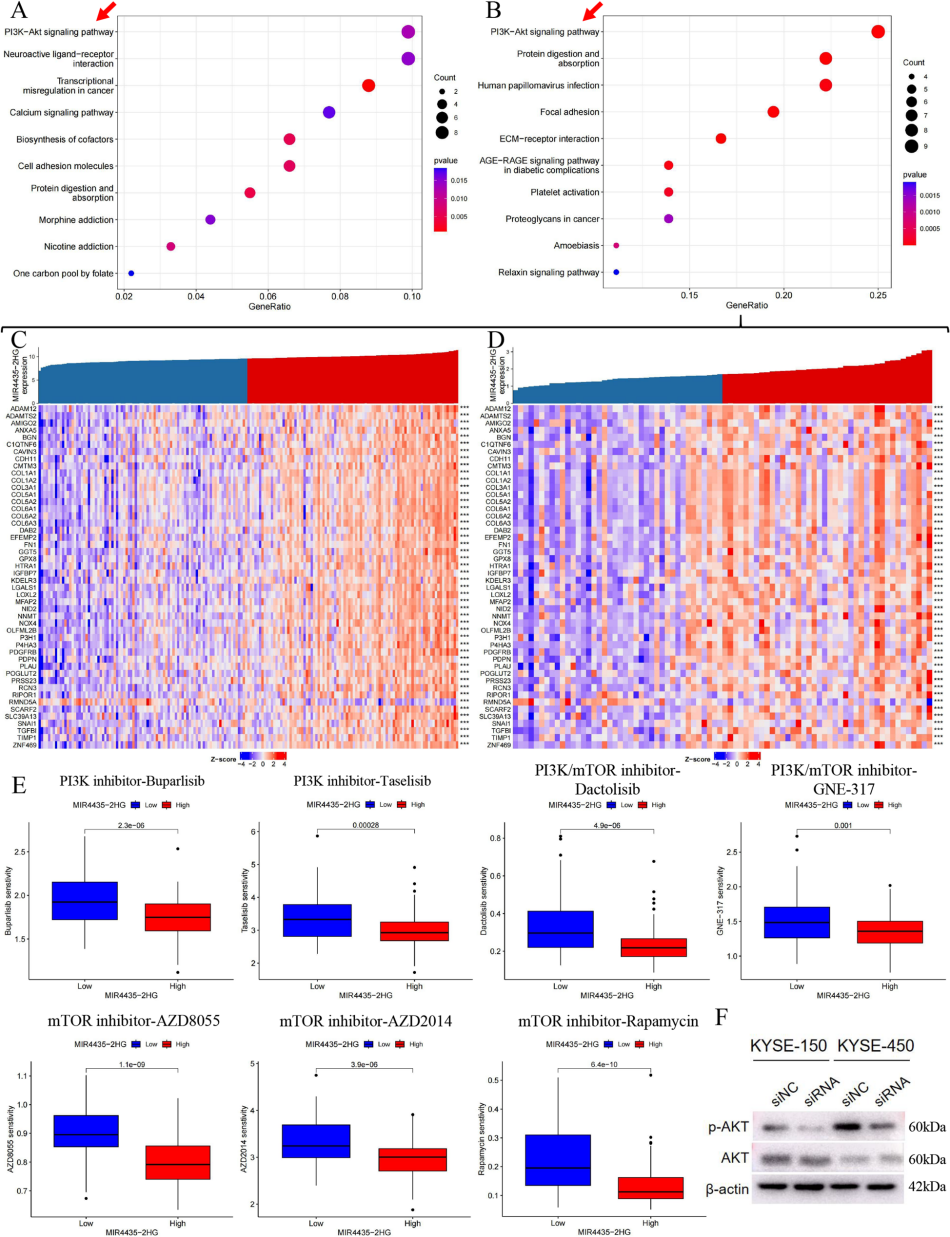
Drug sensitivity and KEGG analysis of MIR4435-2HG. **A** KEGG enrichment analysis showing the PI3K-AKT signaling pathway as the most enriched pathway among the target mRNAs in the ceRNA network. **B** KEGG enrichment analysis of co-expression mRNA indicating that the enrichment degree of PI3K-AKT pathway is the highest. **C**-**D** Heatmap of co-expression relationships of MIR4435-2HG in TCGA-ESCC and GSE53625 datasets. **E** Drug sensitivity analysis demonstrating that groups with high MIR4435-2HG expression are more sensitive to PI3K-AKT pathway inhibitors. **F** Changes of proteins related to the PI3K-AKT pathway after MIR4435-2HG knockdown. Full-length blots/gels are presented in Supplementary Fig. S4

The KEGG enrichment analysis of 188 target mRNAs within the ceRNA network of MIR4435-2HG revealed that the PI3K-AKT signaling pathway exhibited the highest enrichment level (Fig. 7A). This suggests a potential association between MIR4435-2HG and this pathway. However, to determine whether the elevated expression of MIR4435-2HG promotes or inhibits this signaling pathway, further evidence is required. To investigate this, we conducted a co-expression analysis of MIR4435-2HG in the TCGA-ESCC cohort and the GSE53625 cohort to identify mRNAs that exhibit a co-expression relationship with it. The results showed that MIR4435-2HG had consistent co-expression relationships with numerous mRNAs in both cohorts (Fig. 7C, D), indicating MIR4435-2HG may regulate its target mRNA through a stable and conserved biological mechanism and have a significant role in tumor-related biological processes. Concurrently, enrichment analysis of these co-expressed mRNAs revealed that the PI3K-AKT pathway had the highest level of enrichment. Additionally, the strong positive correlations depicted in Fig. 7C, D indicate that elevated expression of MIR4435-2HG was associated with increased expression of proteins in this pathway, providing indirect evidence that MIR4435-2HG may activate the PI3K-AKT pathway.

In order to further explore the relationship between MIR4435-2HG and the PI3K-AKT pathway, we detected the IC50 values of eight inhibitors related to PI3K-AKT pathway through drug sensitivity analysis, and found that the group with high expression of MIR4435-2HG was more sensitive to these drugs (Fig. 7E). This finding provides further evidence that high expression of MIR4435-2HG could activate PI3K-AKT pathway. The Western Blot results confirmed our hypothesis that phosphorylation of AKT (Protein Kinase B, AKT/PKB) was inhibited after MIR4435-2HG knockdown (Fig. 7F). Overall, the above results suggest that MIR4435-2HG may promote the proliferation and migration of ESCC by activating PI3K-AKT pathway, thereby resulting in early postoperative metastasis and poor prognosis.

## Discussion

Metastasis remains a formidable challenge in oncology, contributing significantly to cancer-related mortality worldwide, particularly in aggressive cancers such as ESCC [13, 14]. Despite advancements in cancer research, the lack of reliable molecular biomarkers for predicting metastasis poses a critical barrier in clinical practice. This study underscores the pivotal role of lncRNA MIR4435-2HG in early metastasis and its association with poor prognosis in patients with ESCC. Importantly, this research marks the first investigation into the relationship between MIR4435-2HG expression levels and clinical outcomes in ESCC patients.

Long Non-Coding RNA MIR4435-2 Host Gene, also known as MIR4435-2HG, is a non-coding RNA closely associated with the occurrence, development, and prognosis of various cancers [15–18]. The integration of multi-omic data and the application of rigorous statistical analyses exemplify best practices in biomarker discovery [19, 20]. The high effect value of MIR4435-2HG in the MESU-related prognostic model suggests that it may be a key determinant of prognosis in ESCC patients. The differential expression of MIR4435-2HG in ESCC tissues, particularly its elevated levels in patients with poor prognosis, underscores its potential utility as a biomarker for early metastasis. Traditional prognostic tools, such as the TNM staging system, often fail to account for the observed heterogeneity in clinical outcomes among patients with similar stages [21]. MIR4435-2HG’s ability to distinguish between ESCC patients with high and low metastasis susceptibility offers a valuable tool for personalized treatment strategies. ESCC patients with high MIR4435-2HG expression may benefit from more aggressive postoperative treatments and closer monitoring, even if their tumors are detected at an early stage. This could potentially improve their clinical outcomes by addressing the high risk of early metastasis, which is not evident from traditional pathological assessments alone.

Integrating MIR4435-2HG into molecular typing schemes for ESCC could significantly enhance the precision of cancer classification and treatment. Our study demonstrates that combining MIR4435-2HG with another lncRNA, CASC15 (Cancer Susceptibility Candidate 15), enables the stratification of ESCC patients into distinct molecular subtypes with varying clinical outcomes. CASC15, located on chromosome 6, plays a significant role in cancers biology by promoting tumor cell proliferation, migration, and epithelial-mesenchymal transition (EMT). Additionally, CASC15 has potential as a diagnostic and prognostic biomarker due to its altered expression in cancer, influencing cancer cell survival and chemotherapy resistance [22, 23]. This dual-gene approach provides a more robust prediction of adverse clinical events compared to the analysis based solely on MIR4435-2HG expression levels. Molecular typing based on MIR4435-2HG and CASC15 facilitates the identification of three distinct patient clusters with different survival rates. Patients in cluster B, characterized by lower expression levels of both lncRNAs, exhibit a survival advantage over those in clusters A and C. This stratification could guide the development of personalized treatment strategies, ensuring that patients receive the most effective therapy based on the molecular characteristics of their tumors.

In addition to its prognostic value, MIR4435-2HG also shows potential as a therapeutic target [16–18]. Cell experiments showed that knocking down MIR4435-2HG significantly inhibited the migration and proliferation abilities of different ESCC cells. LncRNAs function in cancer primarily by regulating gene expression, affecting signaling pathways, and modulating cellular behavior [5, 6]. The upregulation of MIR4435-2HG may promote cancer progression through multiple mechanisms. On one hand, MIR4435-2HG might interact with mRNAs, miRNAs, or proteins to regulate their stability or translation efficiency, thereby influencing cancer cell proliferation, apoptosis, migration, and invasion [24, 25]. On the other hand, MIR4435-2HG may regulate gene expression through epigenetic mechanisms, such as DNA methylation, histone modifications, and chromatin remodeling [26]. Studies have shown that MIR4435-2HG can act as an epigenetic regulator by interacting with histone modification enzyme complexes, thereby modulating the expression state of specific genes [27]. Additionally, MIR4435-2HG may be involved in regulating metabolic pathways in cancer cells, such as glycolysis and oxidative phosphorylation, providing sufficient energy and metabolites to support rapid cell proliferation [28].

To further investigate the regulatory role of MIR4435-2HG in gene expression, we constructed a ceRNA network. This network is critical, as miRNAs are known to play pivotal roles in gene regulation, influencing processes such as cell proliferation, apoptosis, and metastasis [29]. The ability of MIR4435-2HG to sequester miRNAs positions it as a key regulator in the cellular environment, potentially altering the expression of numerous genes involved in cancer pathways. Through the construction of the prognostic sub-network, we identified potential genes likely to impact patient outcomes, such as ST8SIA4 (ST8 Alpha-N-Acetyl-Neuraminide Alpha-2,8-Sialyltransferase 4) and PPFIA1 (PTPRF interacting protein alpha 1). In ESCC, increased sialylation mediated by ST8SIA4 can enhance the metastatic potential of cancer cells by promoting cell adhesion, migration, and invasion through interactions with sialoglycan-binding lectins, such as Siglecs and selectins [30, 31]. PPFIA1 has been more thoroughly studied in the context of ESCC prognosis. High expression levels of PPFIA1 are associated with several adverse clinicopathological features, including deeper tumor invasion, lymph node metastasis, and advanced TNM stage. Patients with higher PPFIA1 expression tend to have poorer overall survival rates, making it a potential independent prognostic factor for ESCC [32]. These key lncRNA-miRNA-mRNA axes in the prognostic sub-network offer insights into the specific pathways and interactions most relevant to cancer prognosis.

MIR4435-2HG appears to facilitate ESCC progression primarily through the activation of the PI3K-Akt signaling pathway. This pathway plays a crucial role in tumor metastasis by regulating processes such as cell proliferation, survival, and migration [33]. Activation of this pathway promotes EMT, which enhances the invasive and migratory capabilities of cancer cells [34]. Additionally, PI3K-AKT pathway can inhibit apoptosis and increase angiogenesis, further facilitating metastatic spread [33]. Dysregulation of this pathway is commonly observed in various cancers and is associated with poor prognosis and increased metastatic potential [35]. The positive correlation between MIR4435-2HG expression and key genes of the PI3K-Akt pathway indicates that MIR4435-2HG may act as an upstream regulator, potentially interacting with other RNAs or proteins to activate this pathway. Our drug sensitivity analysis indicates that patients with high MIR4435-2HG expression are more responsive to PI3K-Akt pathway inhibitors such as buparlisib and dactolisib. This finding not only supports the targeted regulatory relationship between MIR4435-2HG and the PI3K-AKT pathway, but also suggests that combining MIR4435-2HG inhibitors with existing PI3K-Akt pathway inhibitors might enhance therapeutic efficacy and overcome resistance mechanisms.

Given the critical role of MIR4435-2HG in cancer, we hypothesize that it may serve as a novel therapeutic target. Inhibiting the expression or function of MIR4435-2HG might effectively halt cancer cell growth and metastasis, thereby achieving anti-tumor effects. Currently, therapeutic strategies targeting lncRNAs primarily include RNA interference, antisense oligonucleotides (ASO), and small molecule inhibitors. For example, Chen et al. reported that MIR4435-2HG knockdown effectively reduced tumor cell proliferation and migration [36]. Additionally, the application of CRISPR/Cas9 gene editing technology provides new possibilities for targeting MIR4435-2HG [37]. By precisely editing the gene sequence of MIR4435-2HG, specific regulation of its expression can be achieved, thus exerting therapeutic effects. Developing MIR4435-2HG-targeted therapies, either through direct inhibition using small interfering RNAs or ASO or through indirect modulation of its regulatory ceRNA network, holds significant promise for treating metastatic ESCC.

Notwithstanding the therapeutic relevance of our findings, several limitations should be acknowledged. First, validating the clinical utility of MIR4435-2HG as a prognostic biomarker in larger, independent cohorts is essential. Prospective studies should assess its performance in predicting early metastasis and long-term survival across diverse populations and clinical settings. Second, although we demonstrated that siRNA-mediated knockdown of MIR4435-2HG suppressed ESCC cell proliferation and migration, over-expression experiments were not performed. Such assays would provide complementary evidence and further strengthen the functional characterization of MIR4435-2HG. Finally, further elucidating the precise molecular mechanisms by which MIR4435-2HG regulates the PI3K-Akt pathway and other associated signaling networks is crucial. Advanced techniques such as RNA immunoprecipitation sequencing and chromatin isolation by RNA purification sequencing could help identify the specific protein and RNA partners of MIR4435-2HG [38, 39], which would provide deeper insights into its functional roles.

## Conclusion

Our study highlights the pivotal role of MIR4435-2HG in ESCC metastasis and prognosis. By elucidating its involvement in the PI3K-Akt pathway and demonstrating its potential as both a prognostic biomarker and therapeutic target, we provide a foundation for future research and clinical applications. Integrating MIR4435-2HG into molecular typing and treatment strategies holds the promise of significantly improving ESCC management, offering hope for better outcomes for patients with this aggressive cancer. As our understanding of lncRNAs and their complex regulatory networks advances, the prospect of personalized and precise cancer therapies becomes increasingly attainable.

## Supplementary Information

Additional file 1: Materials and methods: The Supplementary Materials and methods, including 2.1 (Construction of the MESU-related prognostic signature), 2.2 (Evaluation of the MESU-related prognostic signature), 2.3 (Differential analysis between high- and low-risk groups), 2.4 (Drug screen), 2.5 (Molecular typing), 2.6 (RNA interference and cell experiment), 2.7 (Construction of ceRNA network), 2.8 (Co-expressed analysis and enrichment analysis), 2.9 (Analysis of drug sensitivity), 2.10 (siRNA transfection), 2.11 (Cell culture and functional assays), 2.12 (Western blotting)

Additional file 2: Supplemental Results: Figure S1. The quality validation of the prognostic model. Figure S2. Verification of prediction accuracy of nomogram. Figure S3. Tumor microenvironment, immune function, and GSEA analysis of the MESU-related prognostic model. Figure S4. Western Blot results showed the phosphorylation of the PI3K-AKT signaling pathway after MIR4435-2HG knockdown. Table S1: The results of drug screen in cMAP by all DEGs. Table S2: Clinical information and prognosis of patients in qPCR validation

## Data Availability

The datasets presented in this study can be found in online repositories (TCGA, GEO, and GTEx). The names of the repositories and accession numbers can be found in the article. Further inquiries can be directed to the corresponding authors.

## Abbreviations

AUC: Area under the curve
cMAP: Connectivity map
DEGs: Differentially expressed genes
DElncs: Differentially expressed long non-coding RNAs
DEmRNAs: Differentially expressed mRNAs
DEmis: Differentially expressed microRNAs
ESCA: Esophageal cancer
ESCC: Esophageal squamous cell carcinoma
EAC: Esophageal adenocarcinoma
GSEA: Gene set enrichment analysis
IC50: Half inhibitory concentration
LASSO: Least absolute shrinkage and selection operator
KM: Kaplan Meier
KEGG: Kyoto Encyclopedia of Genes and Genomes
MESUlncs: Metastasis-susceptibility related lncRNAs
MESUGs: Metastasis-susceptibility related genes
OS: Overall survival
PCA: Principal component analysis
RT-qPCR: Quantitative Real-time PCR
ROC: Receiver operating characteristic
TNM: Tumor Node Metastasis
TIICs: Tumor infiltrating immunocytes
