# Supplementary material for: MIR4435-2HG: a key player in the novel lncRNA prognostic signatures causes early metastasis after tumor resection and poor prognosis for esophageal squamous cell carcinoma
Source: BMC Cancer. 2025 Nov 24;26:77. doi: 10.1186/s12885-025-15299-y (PMC12817812; doi:10.1186/s12885-025-15299-y)
Supplement: Supplementary file 1 — Additional file 1: Materials and methods: The Supplementary Materials and methods, including 2.1 (Construction of the MESU-related prognostic signature), 2.2 (Evaluation of the MESU-related prognostic signature), 2.3 (Differential analysis between high- and low-risk groups), 2.4 (Drug screen), 2.5 (Molecular typing), 2.6 (RNA interference and cell experiment), 2.7 (Construction of ceRNA network), 2.8 (Co-expressed analysis and enrichment analysis), 2.9 (Analysis of drug sensitivity), 2.10 (siRNA transfection), 2.11 (Cell culture and functional assays), 2.12 (Western blotting) [file 12885_2025_15299_MOESM1_ESM.docx]

**Materials and methods**

**2.1 Construction of the MESU-related prognostic signature**

Before constructing the prognostic model, we used an instruction named ‘createDataPartition’ in R package ‘creat’ to randomly divide the patients of GSE53625 into training set and validation set (The radio of patient size was 0.5:0.5). Least absolute shrinkage and selection operator (LASSO) is a biased estimation tool for data with complex collinearity; it can select variables and estimate parameter simultaneously and better solve the multicollinearity problem in regression analysis^[1]^. Thus, we used R package ‘glmnet’ to decrease the gene number so as to avoid over-fitting and select the prognostic signature of mRNA and lncRNA to create the prognostic model. The risk score of every patient was calculated based on the prognostic signature, and all patients were separated to high- and low-risk groups with the median score as the cut-off value. Risk score was calculated as follows: Risk score = $\sum_{i=1}^{n} Coefi*expi$, where ‘coef’ was the risk coefficient and ‘exp’ was the expression level of each MESUG.

**2.2 Evaluation of the MESU-related prognostic signature**

R package ‘ggplot2’ and ‘scatterplot3d’ was used to respectively draw principal component analysis (PCA), which could assess the efficiency of cohort clustering. Survival analysis for each group was evaluated by the Kaplan Meier (KM) curve. The receiver operating characteristic (ROC) curve and the area under the curve (AUC) were drawn using R package ‘timeROC’ in R-language. To verify whether the model could be used as an independent clinical prognostic predictor, we integrated the risk score with existing clinicopathological characteristics for univariate and multivariate Cox regression, correlation and heatmap analyses by R package ‘ggpubr’, ‘limma’ ‘ComplexHeatmap’, and ‘RColorBrewer’. Age, gender, loation, grade, stage and risk score were used to construct a nomogram based on R package ‘rms’. The calibration curve was plotted to evaluate the offset between the nomogram and the actual incidence.

**2.3 Differential analysis between** **high- and low-risk groups**

The ESTIMATE algorithm was used to compute the immune, stromal, and ESTIMATE score of each ESCC patient. Then, we utilized CIBERSORT algorithm to estimate the proportion of 22 tumor infiltrating immunocytes (TIICs), and the sum of ratio was 1. The difference analysis of immune function was completed with the help of R package ‘GSVA’ and ‘GSEABase’. Gene set enrichment analysis (GSEA) was carried out for the high- and low-risk groups by using the GSEA_4.3.2 software, with c2.cp.kegg.v2023.1.Hs.symbols.g mt as gene sets database, and the number of stochastic combinations was set to 1000.

**2.4 Drug screen**

Potential small molecule drugs for postoperative metastasis were forecasted using the connectivity map (cMAP, https://clue.io/), which incorporates genome-wide transcriptional expression data from small molecule drugs. The symbols of DEG from the high- and low-risk groups were inputted to search for drugs that could counteract the changes of gene expression. A negative score indicates that small molecule compounds or drugs have an antagonistic relationship with the biological processes or states of differential genes. This suggests that these genes and drugs have opposite mechanisms of action and may represent potential therapeutic options for ESCC metastasis.

**2.5 Molecular typing**

The number of unsupervised clusters and their stability were estimated using the consensus clustering method based on the MIR4435-2HG and CASC15 expression profiles (the results of RT-qPCR). The principal component analysis (PCA), employed to assess the efficiency of cohort clustering, was completed using the R package ‘ggplot2’. Other analytical methods, including the KM curve and correlation heatmap, remained consistent with those described in section **2.3** of this manuscript.

**2.6 RNA interference and cell experiment**

Human esophageal squamous cell carcinoma (ESCC) cell line KYSE-510 were obtained from Cobioer Bioscience (Nanjing, China), while ESCC cell line KYSE-140 were obtained from Cell Bank of the Typical Culture Preservation Committee of the Chinese Academy of Sciences (Shanghai, China). KYSE-510 and KYSE-140 were grown in RPMI 1640 (R10-040-cv-1803, Corning, Beijing, China), supplemented with 10% fetal bovine serum (FBS) (04-001-1A, VivaCell, Shanghai, China) and 1% penicillin-streptomycin solution (P/S) (P1400, Solarbio, Beijing, China). MIR4435-2HG inhibitor siRNAs were commercially synthesized by Genepharma (Suzhou, China). All transfections were performed using Lipofectamine 2000 (Invitrogen, USA), with transfection efficiency assessed by RT-qPCR analysis. Subsequent experiments were performed at 48 h post transfection. The transfected cells were cultured in 96-well plates at a density of 2 × 10^4^ cells per well and incubated for 24, 48, and 72 h. Cell proliferation was assayed using the MTT assay (Beyotime, China) according to the manufacturer’s protocol. Migration assays were performed using 8 μm Transwell chambers (Corning Company, NY, USA). Following transfection, the cells were planted into the upper chambers (1 × 10^5^ cells/well) and cultured for 24 h (KYSE150) and 36 h (KYSE450). The chambers were then fixed with 4% paraformaldehyde and stained with crystal violet for 8 min. Stained areas were quantified using ImageJ after washing out the crystal violet.

**2.7 Construction of ceRNA network**

LncBase (https://diana.e-ce.uth.gr/lncbasev3/home) was utilized to identify the target miRNAs of MIR4435-2HG, and the relationships between mRNA and miRNA in the ceRNA network were defined using TargetScan and miRDB. The differentially expressed miRNAs (DEmis) in the GSE97051 dataset were identified using the ‘limma’ package in R (*P* < 0.05 and │log2 fold change (FC)│ > 0.585), optimizing the lncRNA-miRNA network. The DEGs in our RNA-seq data were identified using the ‘limma’ package in R (*P* < 0.05 and │log2 fold change (FC)│ > 1), optimizing the miRNA-mRNA network. Univariate COX regression analysis was used to identify prognostic mRNAs, further refining the prognostic sub-network in the ceRNA network. The MIR4435-2HG-miRNA-mRNA network was constructed using Cytoscape (version 3.8.0).

**2.8 Co-expressed analysis and enrichment analysis**

According to Pearson’ s correlation analysis, the corresponding mRNAs in both the TCGA database and GSE53625 dataset were selected, with an absolute value of the correlation coefficient greater than 0.4 and *P* < 0.001). Using the R package ‘clusterprofiler’, we conducted Kyoto Encyclopedia of Genes and Genome (KEGG) pathway analyses for the mRNAs involved in the constructed ceRNA network and exhibiting co-expression with MIR4435-2HG. The analysis was conducted with a set of cut-off criteria at P < 0.05.

**2.9 Analysis of drug sensitivity**

We utilized drug sensitivity analysis to predict the half inhibitory concentration (IC50) values of the PI3K-AKT signaling pathway inhibitors using the R package ‘oncoPredict’, including drugs such as buparlisib, taselisib, dactolisib, and others.

**2.10 siRNA transfection**

To knock down MIR4435-2HG, small interfering RNA (siRNA) targeting MIR4435-2HG (5′-CATTGCATAATGGATGGCTTGTCCT-3′) and a nonspecific negative control siRNA were synthesized by GenePharma (Shanghai, China). KYSE-150 and KYSE-450 cells were seeded into six-well plates and transfected when cell confluence reached approximately 30% using Lipofectamine 2000 (Invitrogen, USA) according to the manufacturer’s instructions. After 48h of incubation, cells were harvested for RNA extraction or functional assays to evaluate knockdown efficiency.

**2.11 Cell culture and functional assays**

Human esophageal squamous cell carcinoma cell lines KYSE-150 and KYSE-450 were cultured in RPMI-1640 medium supplemented with 10% fetal bovine serum and 1% penicillin–streptomycin at 37℃ in a humidified incubator with 5% CO₂. Transwell chambers with 8-µm-pore-size membranes (353097, Corning, New York, NY, USA) were used for the Transwell assay. A total of 1 × 10^5^ cells in serum-free medium were seeded into the upper chamber, and 650 µL of complete medium was placed in the lower chamber as a chemoattractant. After 24h of incubation, migrated cells on the lower membrane surface were fixed and stained with crystal violet. Six high-power fields (HPFs) per insert were imaged at a fixed magnification, and the stained coverage area (migrated area fraction) was quantified with ImageJ. For the MTT assay, 2 × 10^3^ cells per well were seeded into 96-well plates (six replicate wells per condition), cultured for up to 72h, treated with MTT, and cell viability was assessed by measuring absorbance at 490 nm with a microplate reader; proliferation was calculated from changes in A_490_ over time.

**2.12 Western blotting**

Total protein was extracted from KYSE150 and KYSE450 cells using RIPA lysis buffer (Beyotime, Shanghai, China) supplemented with protease and phosphatase inhibitors. Protein concentrations were determined with a BCA assay kit (Thermo Fisher Scientific, USA). Equal amounts of protein (20 μg per lane) were separated by 10% SDS-PAGE and transferred onto PVDF membranes (Millipore, USA). Membranes were blocked with 5% non-fat milk in TBST for 1 h at room temperature and then incubated overnight at 4 °C with the following primary antibodies: anti-PI3K (1:1000, Cell Signaling Technology), anti-AKT (1:1000, CST), anti-p-AKT (Ser473, 1:1000, CST), and anti-GAPDH (1:2000, Proteintech). After washing, membranes were incubated with HRP-conjugated secondary antibodies (1:5000, CST) for 1 h at room temperature. Protein bands were visualized using an enhanced chemiluminescence (ECL) kit (Millipore) and imaged with a ChemiDoc imaging system (Bio-Rad, USA). The molecular weight markers were included and the apparent molecular weights of protein bands were indicated in the revised figures.
