# Supplementary material for: MIR4435-2HG: a key player in the novel lncRNA prognostic signatures causes early metastasis after tumor resection and poor prognosis for esophageal squamous cell carcinoma
Source: BMC Cancer. 2025 Nov 24;26:77. doi: 10.1186/s12885-025-15299-y (PMC12817812; doi:10.1186/s12885-025-15299-y)
Supplement: Supplementary file 2 — Additional file 2: Supplemental Results: Figure S1. The quality validation of the prognostic model. Figure S2. Verification of prediction accuracy of nomogram. Figure S3. Tumor microenvironment, immune function, and GSEA analysis of the MESU-related prognostic model. Figure S4. Western Blot results showed the phosphorylation of the PI3K-AKT signaling pathway after MIR4435-2HG knockdown. Table S1: The results of drug screen in cMAP by all DEGs. Table S2: Clinical information and prognosis of patients in qPCR validation [file 12885_2025_15299_MOESM2_ESM.docx]

**Supplementary figure1:**

**
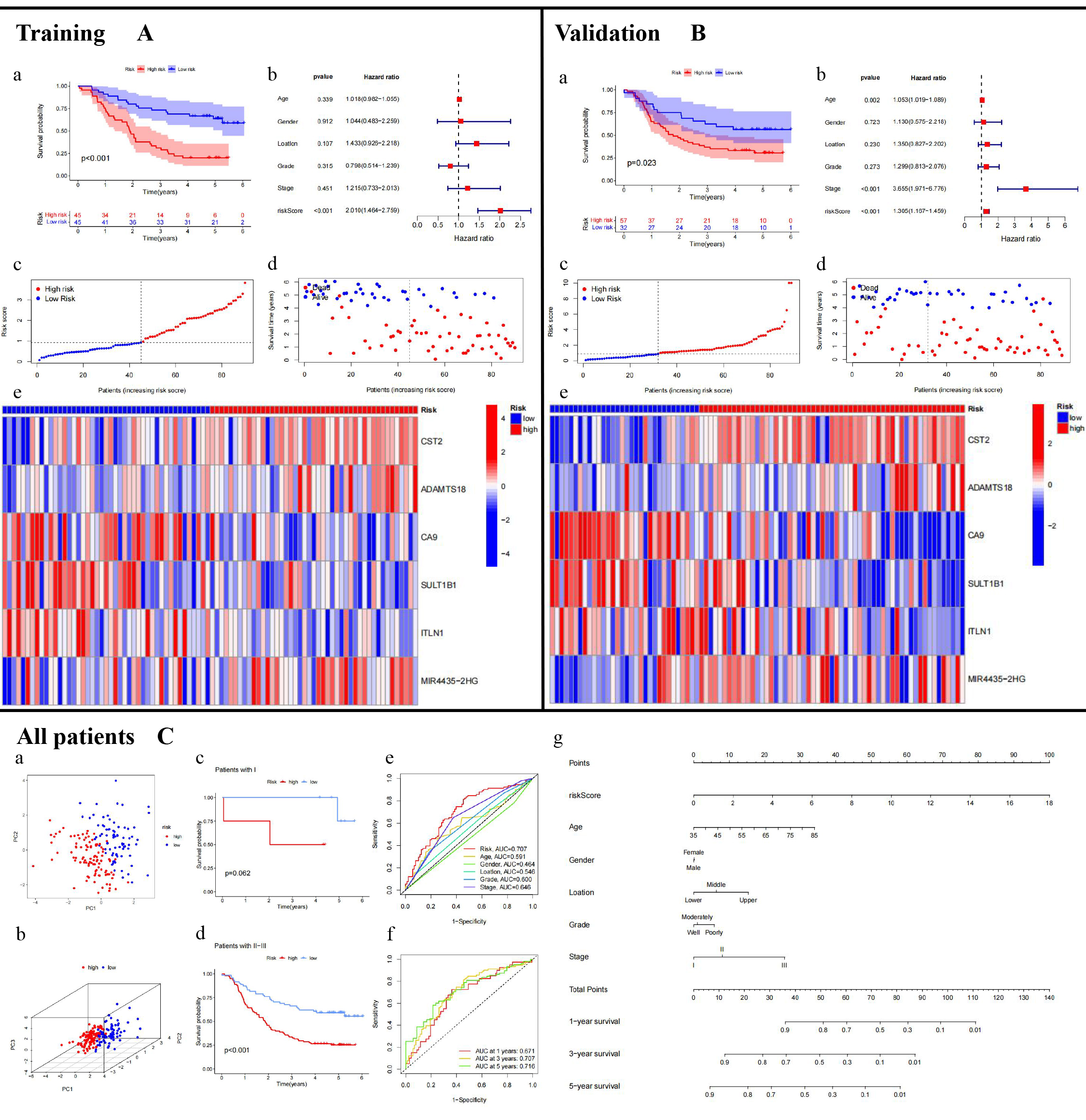
**

**Figure S1.** The quality validation of the prognostic model. (A and B-a) KM survival curves of the high- and low-risk groups in training set and validation set. (A and B-b) Forest plots show significant survival-related clinicopathological features following multivariate Cox regression analysis. (A and B-c to e) The distribution of risk scores (c), survival times/status (d), and gene expression levels (e). (C-a and b) The PCA and three-dimensional PCA plots of different risk groups based on the MESU-related prognostic model. (C-c and d) KM survival curves of the high- and low-risk groups in stage I patients and stage II-III patients. (C-e) Multi-index ROC curves of age, gender, stage, grade, location and risk Score. (C-f) Multi-time ROC curves of 1, 3, 5 years. (C-g) Nomogram of the prognostic model.

**Supplementary figure2:**


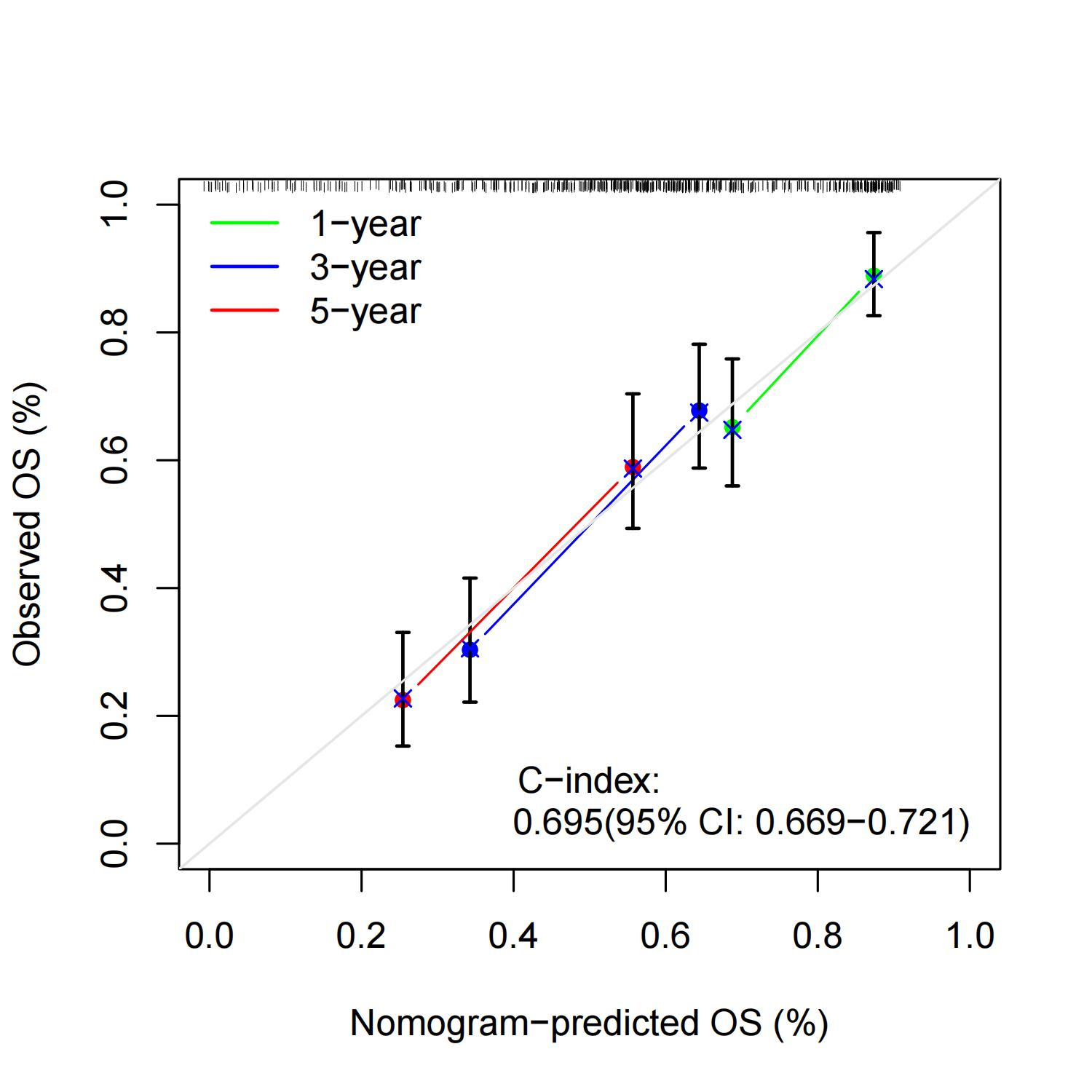


**Figure S2.** Verification of prediction accuracy of nomogram.

**Supplementary figure3:**

**
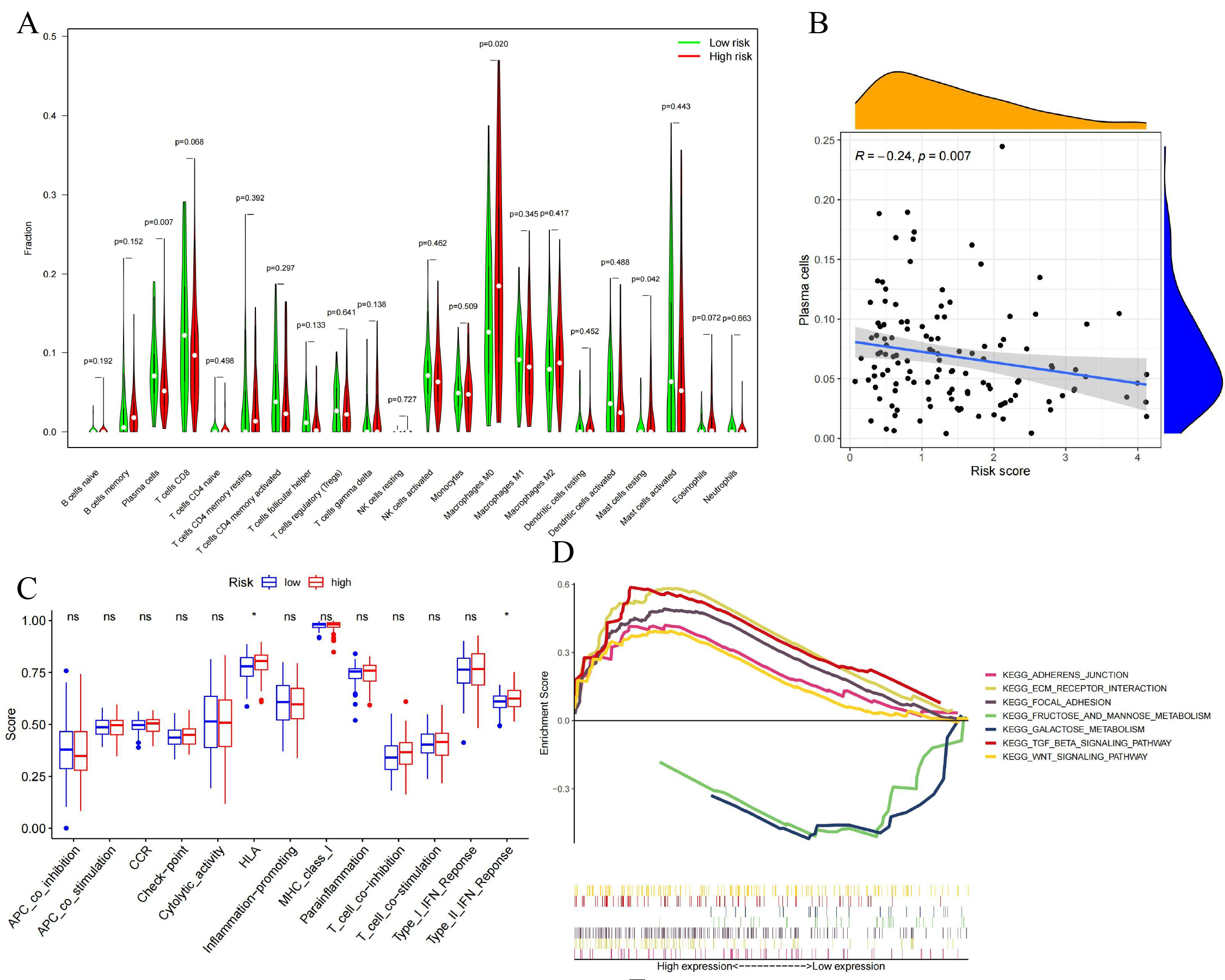
**

**Figure S3.** Tumor microenvironment, immune function, and GSEA analysis of the MESU-related prognostic model. (A) Immune cell infiltration in high-risk (red) and low-risk (green) groups. *P*-values indicate the statistical significance of these differences. (B) Correlation between the risk score and plasma cell levels, with a negative correlation (R = -0.24, *P* = 0.007). (C) Box plots comparing immune function activities between high-risk (red) and low-risk (blue) groups. (D) Gene Set Enrichment Analysis (GSEA) highlighting pathways enriched in high-risk patients. Significant pathways include KEGG_ADHERENS_JUNCTION, KEGG_FOCAL_ADHESION, KEGG_ECM_RECEPTOR_INTERACTION and so on, which are associated with cancer progression and metastasis.

**
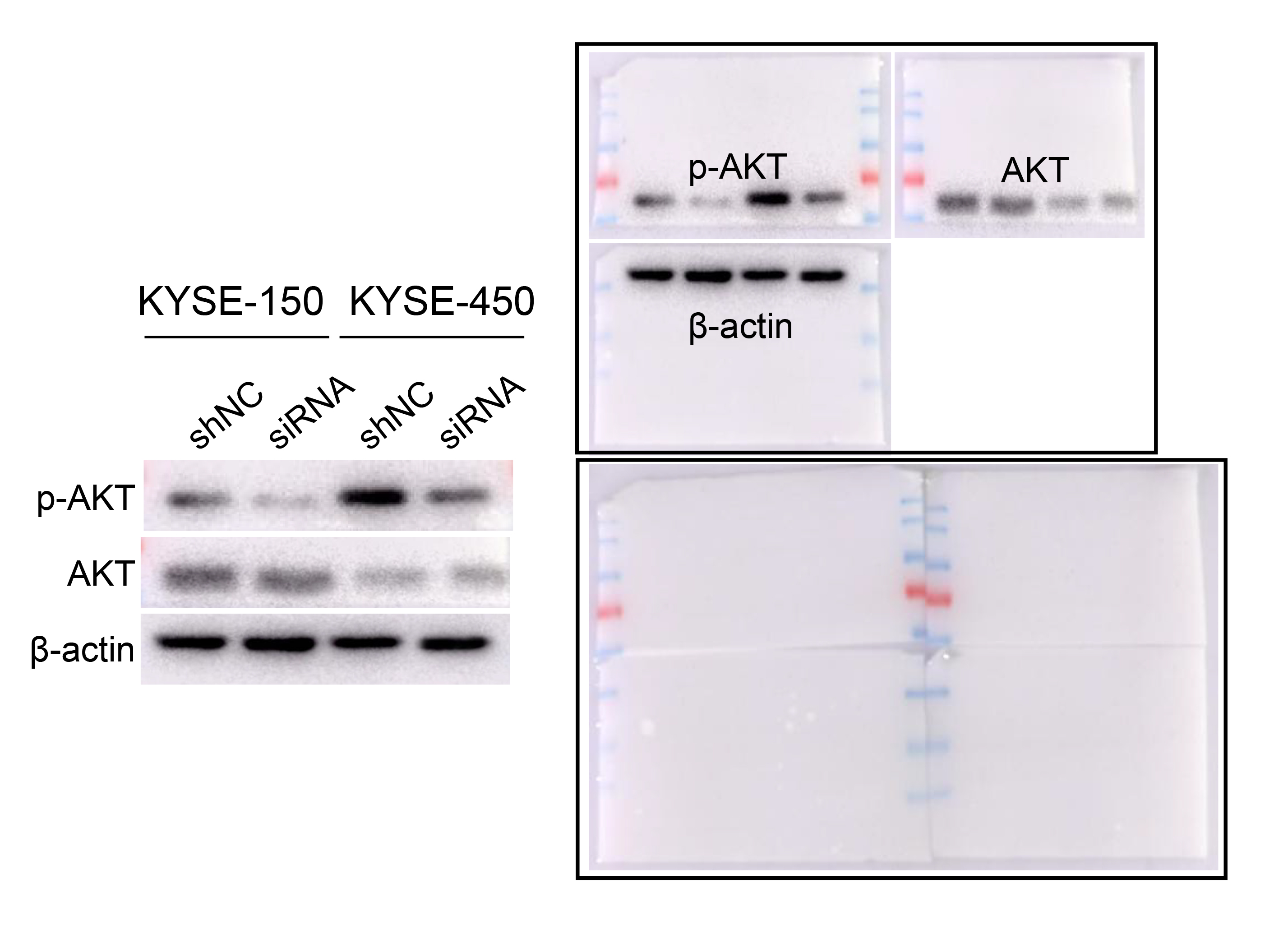
**

**Figure S4.** Western Blot results showed the phosphorylation of the PI3K-AKT signaling pathway after MIR4435-2HG knockdown.

**Supplementary table1:**

| Table S1: The results of drug screen in cMAP by all DEGs. | | | | |
| --- | --- | --- | --- | --- |
| **Rank** | **Score** | **ID** | **Name** | **Description** |
| 8546 | -98.34 | BRD-K59184148 | SB-216763 | Glycogen synthase kinase inhibitor |
| 8543 | -98.03 | BRD-K15402119 | huperzine-a | Acetylcholinesterase inhibitor |
| 8533 | -96.32 | BRD-K37846922 | 3,3'-diindolylmethane | CHK inhibitor |
| 8532 | -96.31 | BRD-K85503079 | perospirone | Dopamine receptor antagonist |
| 8531 | -96.05 | BRD-K57011718 | UK-356618 | Metalloproteinase inhibitor |
| 8529 | -95.84 | BRD-K16508793 | diazepam | Benzodiazepine receptor agonist |
| 8528 | -95.77 | BRD-K68867920 | quetiapine | Dopamine receptor antagonist |
| 8523 | -95.01 | BRD-K84266862 | BRL-50481 | Phosphodiesterase inhibitor |
| 8512 | -92.85 | BRD-K28120222 | parthenolide | NFkB pathway inhibitor |
| 8511 | -92.84 | BRD-K96720755 | relcovaptan | Vasopressin receptor antagonist |
| 8500 | -90.78 | BRD-K57926513 | tyrphostin-AG-1295 | PDGFR receptor inhibitor |
| 8496 | -90.23 | BRD-A52650764 | ingenol | PKC activator |
| 8491 | -89.91 | BRD-K06878038 | deferiprone | Chelating agent |
| 8490 | -89.83 | BRD-K03981224 | ethisterone | Progestogen hormone |
| 8489 | -89.67 | BRD-K77998258 | ganglioside | SRC activator |
| 8487 | -89.5 | BRD-K56450366 | NSC-94258 | Antineoplastic |
| 8483 | -89.21 | BRD-A15914070 | 4-hydroxy-2-nonenal | Cytotoxic lipid peroxidation product |
| 8482 | -89.15 | BRD-K92991072 | PAC-1 | Caspase activator |
| 8480 | -89.12 | BRD-K76205745 | losartan | Angiotensin receptor antagonist |
| 8479 | -88.89 | BRD-K63630713 | etacrynic-acid | Sodium/potassium/chloride transporter inhibitor |
| 8478 | -88.81 | BRD-K35687265 | ON-01910 | PLK inhibitor |
| 8477 | -88.67 | BRD-K62965247 | tipifarnib-P2 | farnesyltransferase inhibitor |
| 8476 | -88.56 | BRD-K22631935 | neurodazine | Neurogenesis of non-pluripotent C2C12 myoblast inducer |
| 8474 | -88.38 | BRD-A74771556 | nikkomycin | Chitin inhibitor |
| 8470 | -87.74 | BRD-K05658747 | raltegravir | HIV integrase inhibitor |
| 8469 | -87.67 | BRD-K96263742 | GW-7647 | PPAR receptor agonist |
| 8465 | -86.9 | BRD-K48427617 | U-0124 | MEK inhibitor |
| 8455 | -85.59 | BRD-K59753975 | vindesine | Tubulin inhibitor |
| 8451 | -85.16 | BRD-K86003836 | flubendazole | Tubulin inhibitor |
| 8448 | -84.67 | BRD-K81376179 | TCS-359 | FLT3 inhibitor |
| 8447 | -84.61 | BRD-K56614220 | clofazimine | GK0582 inhibitor |
| 8445 | -84.31 | BRD-K98548675 | parthenolide | NFkB pathway inhibitor |
| 8444 | -84.09 | BRD-A36267905 | buphenine | Adrenergic receptor agonist |
| 8442 | -84 | BRD-K32107296 | temozolomide | DNA alkylating agent |
| 8439 | -83.85 | BRD-K26521938 | dinoprostone | Prostanoid receptor agonist |
| 8436 | -83.61 | BRD-K74112339 | acetohydroxamic-acid | Urease inhibitor |
| 8435 | -83.51 | BRD-K50311478 | tosyl-phenylalanyl-chloromethyl-ketone | Chymotrypsin inhibitor |
| 8431 | -82.99 | BRD-K13571841 | pepstatin | Aspartic protease inhibitor |
| 8427 | -82.84 | BRD-K93258693 | GW-9662 | PPAR receptor antagonist |
| 8422 | -81.18 | BRD-K91145395 | prostratin | PKC activator |
| 8419 | -80.74 | BRD-K28360340 | TW-37 | BCL inhibitor |
| 8414 | -80.36 | BRD-K49519144 | LY-2140023 | Glutamate receptor agonist |

**Supplementary table2:**

| **Table S2**: Clinical information and prognosis of patients in qPCR validation. | | | | | | | | | | | | |
| --- | --- | --- | --- | --- | --- | --- | --- | --- | --- | --- | --- | --- |
| **ID** | **Surgery Date** | **Gender** | **Age (year)** | **Stage** | **T** | **N** | **Tumor size(cm)** | **Tumor location** | **Tumor type** | **Differentiation degree** | **Anastomoses methods** | **Overall survival(mouth)** |
| 374 | 2014.01.10 | Male | 50 | ⅢB | T3 | N1 | 5.1 | upper esophagus | ulcerative type | middle | instrument anastomosis(neck) | 14 |
| 400 | 2014.03.27 | Female | 67 | ⅢA | T2 | N1 | 8.3 | lower esophagus | ulcerative type | middle | esophagogastrostomy below aortic arch(surgicalstapler) | 6 |
| 447 | 2014.05.05 | Male | 64 | ⅡB | T3 | N0 | 6.2 | middle esophagus | medullary type | middle | esophagogastrostomy over aortic arch(surgicalstapler) | 9 |
| 450 | 2014.05.06 | Male | 59 | ⅡA | T2 | N0 | 10 | lower esophagus | medullary type | middle | esophagogastrostomy below aortic arch(surgicalstapler) | 12 |
| 463 | 2014.05.31 | Male | 63 | ⅠB | T2 | N0 | 5.6 | middle esophagus | ulcerative type | high | esophagogastrostomy over aortic arch(surgicalstapler) | 11 |
| 481 | 2014.07.15 | Female | 71 | ⅡB | T3 | N0 | 6.3 | middle esophagus | medullary type | middle-high | esophagogastrostomy over aortic arch(surgicalstapler) | 7 |
| 509 | 2014.09.04 | Female | 72 | ⅡA | T2 | N0 | 5.6 | middle esophagus | ulcerative type | middle | esophagogastrostomy over aortic arch(surgicalstapler) | 6 |
| 511 | 2014.09.15 | Male | 67 | ⅢB | T3 | N1 | 8.4 | middle esophagus | ulcerative type | high | esophagogastrostomy over aortic arch(surgicalstapler) | 11 |
| 517 | 2014.09.25 | Male | 76 | ⅢB | T3 | N1 | 4.8 | middle esophagus | protruded type | middle | esophagogastrostomy over aortic arch(surgicalstapler) | 12 |
| 518 | 2014.09.26 | Male | 69 | ⅢB | T3 | N1 | 5 | middle esophagus | constritive type | low | esophagogastrostomy over aortic arch(surgicalstapler) | 13 |
| 526 | 2014.10.16 | Male | 71 | ⅢB | T3 | N2 | 3.6 | lower esophagus | medullary type | low | esophagogastrostomy below aortic arch(surgicalstapler) | 12 |
| 528 | 2014.10.18 | Male | 60 | ⅢB | T3 | N2 | 6.1 | middle esophagus | medullary type | middle | esophagogastrostomy over aortic arch(surgicalstapler) | 14 |
| 531 | 2014.10.21 | Male | 63 | ⅢB | T3 | N1 | 3 | middle esophagus | ulcerative type | high | esophagogastrostomy over aortic arch(surgicalstapler) | 14 |
| 542 | 2014.11.08 | Female | 66 | ⅢB | T3 | N2 | 5.4 | middle esophagus | medullary type | middle | esophagogastrostomy over aortic arch(surgicalstapler) | 13 |
| 543 | 2014.11.10 | Male | 65 | ⅢB | T3 | N1 | 5 | middle esophagus | ulcerative type | high | esophagogastrostomy over aortic arch(surgicalstapler) | 13 |
| 548 | 2014.11.19 | Male | 66 | ⅡB | T3 | N0 | 4.7 | upper esophagus | ulcerative type | middle | esophagogastrostomy over aortic arch(surgicalstapler) | 13 |
| 551 | 2014.11.25 | Female | 65 | ⅢB | T3 | N1 | 5 | middle esophagus | ulcerative type | high | esophagogastrostomy over aortic arch(surgicalstapler) | 10 |
| 558 | 2014.12.17 | Male | 61 | ⅡA | T3 | N0 | 5 | lower esophagus | ulcerative type | middle | esophagogastrostomy over aortic arch(surgicalstapler) | 12 |
| 586 | 2015.02.11 | Male | 73 | ⅢB | T3 | N1 | 8 | middle esophagus | medullary type | high | esophagogastrostomy over aortic arch(surgicalstapler) | 10 |
| 595 | 2015.03.17 | Female | 72 | ⅢB | T3 | N2 | 8.7 | middle esophagus | ulcerative type | middle-low | manual anastomosis(neck) | 6 |
| 611 | 2015.04.23 | Male | 62 | ⅠB | T2 | N0 | 7 | middle esophagus | ulcerative type | high | manual anastomosis(neck) | 12 |
| 613 | 2015.04.25 | Male | 76 | ⅡB | T3 | N0 | 3.3 | middle esophagus | ulcerative type | low | esophagogastrostomy over aortic arch(surgicalstapler) | 8 |
| 639 | 2015.06.25 | Male | 64 | ⅡA | T3 | N0 | 7 | middle esophagus | ulcerative type | high | esophagogastrostomy over aortic arch(surgicalstapler) | 9 |
| 644 | 2015.07.07 | Male | 52 | ⅡB | T3 | N0 | 5 | middle esophagus | medullary type | middle | instrument anastomosis(neck) | 7 |
| 653 | 2015.08.06 | Male | 59 | ⅡA | T3 | N0 | 5 | middle esophagus | ulcerative type | high | esophagogastrostomy over aortic arch(surgicalstapler) | 9 |
| 680 | 2015.10.19 | Male | 56 | ⅡA | T2 | N0 | 3.2 | lower esophagus | medullary type | low | esophagogastrostomy below aortic arch(surgicalstapler) | 13 |
| 693 | 2015.11.26 | Male | 72 | ⅢB | T3 | N2 | 5 | middle esophagus | ulcerative type | middle | esophagogastrostomy over aortic arch(surgicalstapler) | 4 |
| 699 | 2015.12.16 | Male | 80 | ⅢB | T3 | N1 | 5.2 | lower esophagus | ulcerative type | middle-low | esophagogastrostomy over aortic arch(surgicalstapler) | 4 |
| 717 | 2016.02.01 | Male | 62 | ⅡA | T3 | N0 | 5 | middle esophagus | medullary type | high | esophagogastrostomy over aortic arch(surgicalstapler) | 6 |
| 744 | 2016.04.05 | Male | 52 | ⅡB | T3 | N0 | 8.9 | middle esophagus | ulcerative type | middle-high | esophagogastrostomy over aortic arch(surgicalstapler) | 7 |
| 745 | 2016.04.13 | Male | 68 | ⅡA | T3 | N0 | 6.9 | middle esophagus | ulcerative type | high | esophagogastrostomy over aortic arch(surgicalstapler) | 12 |
| 753 | 2016.04.25 | Female | 70 | ⅠB | T1b | N0 | 2.7 | middle esophagus | medullary type | middle-low | esophagogastrostomy over aortic arch(surgicalstapler) | 6 |
| 752 | 2016.04.25 | Male | 68 | ⅢB | T3 | N1 | 2.4 | lower esophagus | ulcerative type | middle | esophagogastrostomy over aortic arch(surgicalstapler) | 6 |
| 759 | 2016.05.10 | Male | 73 | ⅡB | T3 | N0 | 5.1 | middle esophagus | medullary type | middle | manual anastomosis(neck) | 11 |
| 771 | 2016.06.20 | Male | 64 | ⅡA | T3 | N0 | 9 | middle esophagus | ulcerative type | high | esophagogastrostomy over aortic arch(surgicalstapler) | 12 |
| 786 | 2016.08.10 | Male | 69 | ⅢB | T3 | N2 | 7.2 | lower esophagus | ulcerative type | high | esophagogastrostomy over aortic arch(surgicalstapler) | 7 |
| 814 | 2016.11.01 | Male | 68 | ⅡB | T3 | N0 | 8.3 | upper esophagus | medullary type | middle | esophagogastrostomy over aortic arch(surgicalstapler) | 9 |
| 815 | 2016.11.14 | Male | 60 | ⅢB | T3 | N2 | 8.3 | middle esophagus | ulcerative type | low | esophagogastrostomy over aortic arch(surgicalstapler) | 5 |
| 825 | 2016.12.12 | Male | 61 | ⅢB | T3 | N1 | 8.4 | middle esophagus | ulcerative type | high | esophagogastrostomy over aortic arch(surgicalstapler) | 10 |
| 828 | 2016.12.17 | Male | 67 | ⅢB | T3 | N1 | 7 | middle esophagus | ulcerative type | high | esophagogastrostomy over aortic arch(surgicalstapler) | 9 |
| 837 | 2017.01.14 | Male | 66 | ⅢB | T3 | N1 | 5.8 | middle esophagus | medullary type | middle-high | esophagogastrostomy over aortic arch(surgicalstapler) | 10 |
| 852 | 2017.02.23 | Male | 61 | ⅡA | T3 | N0 | 7.2 | upper esophagus | medullary type | high | manual anastomosis(neck) | 12 |
| 866 | 2017.03.30 | Male | 61 | ⅢB | T3 | N1 | 4 | lower esophagus | medullary type | low | esophagogastrostomy below aortic arch(surgicalstapler) | 11 |
| 927 | 2017.09.13 | Male | 71 | ⅢB | T3 | N1 | 4.3 | lower esophagus | mushroom type | middle-low | esophagogastrostomy over aortic arch(surgicalstapler) | 7 |
| 947 | 2017.11.23 | Male | 74 | ⅢB | T3 | N1 | 9.7 | middle esophagus | ulcerative type | middle | esophagogastrostomy over aortic arch(surgicalstapler) | 6 |
| 957 | 2017.12.14 | Female | 60 | ⅢB | yT3 | N2 | 4.2 | middle esophagus | ulcerative type | middle | instrument anastomosis(neck) | 7 |
| **ID** | **Surgery Date** | **Gender** | **Age (year)** | **Stage** | **T** | **N** | **Tumor size(cm)** | **Tumor location** | **Tumor type** | **Differentiation degree** | **Anastomoses methods** | **Overall survival(year)** |
| 379 | 2014.1.14 | Male | 46 | ⅡB | T3 | N0 | 7.7 | middle esophagus | ulcerative type | middle | esophagogastrostomy over aortic arch(surgicalstapler) | >8 |
| 399 | 2014.2.24 | Male | 65 | ⅠB | T1b | N0 | 4.3 | middle esophagus | medullary type | high | esophagogastrostomy over aortic arch(surgicalstapler) | >8 |
| 404 | 2014.3.7 | Female | 77 | ⅡB | T3 | N0 | 3.5 | middle esophagus | medullary type | low | esophagogastrostomy over aortic arch(surgicalstapler) | >8 |
| 415 | 2014.3.24 | Male | 64 | ⅠB | T1b | N0 | 2.6 | middle esophagus | ulcerative type | high | esophagogastrostomy over aortic arch(surgicalstapler) | 5 |
| 426 | 2014.4.3 | Male | 61 | ⅠB | T1b | N0 | 0.5 | middle esophagus | erosive type | high | esophagogastrostomy over aortic arch(surgicalstapler) | >8 |
| 428 | 2014.4.12 | Female | 67 | ⅡA | T3 | N0 | 4.6 | middle esophagus | medullary type | high | esophagogastrostomy over aortic arch(surgicalstapler) | >8 |
| 431 | 2014.4.15 | Male | 61 | ⅡA | T3 | N0 | 5.9 | middle esophagus | ulcerative type | high | esophagogastrostomy over aortic arch(surgicalstapler) | 7 (87 mouths) |
| 439 | 2014.4.25 | Male | 67 | ⅠB | T2 | N0 | 5 | middle esophagus | medullary type | high | esophagogastrostomy over aortic arch(surgicalstapler) | >8 |
| 443 | 2014.4.28 | Male | 57 | ⅡA | T3 | N0 | 4.7 | lower esophagus | medullary type | high | esophagogastrostomy below aortic arch(surgicalstapler) | >8 |
| 466 | 2014.6.4 | Female | 59 | ⅠB | T1a | N0 | 3.9 | middle esophagus | mushroom type | middle-low | esophagogastrostomy over aortic arch(surgicalstapler) | >7 |
| 467 | 2014.6.9 | Male | 70 | ⅠB | T2 | N0 | 4.5 | lower esophagus | medullary type | high | esophagogastrostomy below aortic arch(surgicalstapler) | 7 (95 mouths) |
| 470 | 2014.6.14 | Male | 57 | ⅡB | T3 | N0 | 2 | middle esophagus | medullary type | middle | esophagogastrostomy over aortic arch(surgicalstapler) | >7 |
| 473 | 2014.6.28 | Male | 62 | ⅡB | T3 | N0 | 5 | upper esophagus | medullary type | middle | esophagogastrostomy over aortic arch(surgicalstapler) | >7 |
| 484 | 2014.7.21 | Male | 64 | ⅠB | T2 | N0 | 3.8 | lower esophagus | ulcerative type | high | esophagogastrostomy below aortic arch(surgicalstapler) | >7 |
| 486 | 2014.7.22 | Male | 72 | ⅡB | T3 | N0 | 12.8 | middle esophagus | medullary type | low | manual anastomosis(neck) | >7 |
| 491 | 2014.8.4 | Male | 59 | ⅢA | T2 | N1 | 3.5 | lower esophagus | ulcerative type | high | esophagogastrostomy below aortic arch(surgicalstapler) | >7 |
| 500 | 2014.8.21 | Female | 65 | ⅡA | T2 | N0 | 5.3 | middle esophagus | medullary type | middle | esophagogastrostomy over aortic arch(surgicalstapler) | >7 |
| 514 | 2014.9.20 | Female | 65 | ⅠC | yT2 | N0 | 5 | middle esophagus | ulcerative type | high | esophagogastrostomy over aortic arch(surgicalstapler) | >7 |
| 516 | 2014.9.25 | Female | 63 | ⅡB | T3 | N0 | 6.5 | middle esophagus | ulcerative type | middle | esophagogastrostomy over aortic arch(surgicalstapler) | >7 |
| 527 | 2014.10.18 | Male | 68 | ⅠB | T1b | N0 | 5.6 | middle esophagus | protruded type | low | esophagogastrostomy over aortic arch(surgicalstapler) | >7 |
| 529 | 2014.10.20 | Male | 60 | ⅠB | T2 | N0 | 4.3 | middle esophagus | medullary type | high | esophagogastrostomy over aortic arch(surgicalstapler) | >7 |
| 530 | 2014.10.20 | Male | 61 | ⅠB | T2 | N0 | 4.8 | middle esophagus | medullary type | high | esophagogastrostomy over aortic arch(surgicalstapler) | >7 |
| 532 | 2014.10.22 | Male | 66 | ⅠB | T1b | N0 | 3.5 | lower esophagus | protruded type | low | esophagogastrostomy below aortic arch(surgicalstapler) | >7 |
| 537 | 2014.10.30 | Female | 70 | ⅡB | T3 | N0 | 6 | middle esophagus | ulcerative type | middle | Right intrathoracic anastomosis(surgicalstapler) | 5 (62 mouths) |
| 546 | 2014.11.15 | Male | 66 | ⅡA | T3 | N0 | 6.2 | middle esophagus | ulcerative type | high | esophagogastrostomy over aortic arch(surgicalstapler) | >7 |
| 547 | 2014.11.17 | Male | 61 | ⅢA | T2 | N1 | 5.6 | middle esophagus | medullary type | middle | esophagogastrostomy over aortic arch(surgicalstapler) | >7 |
| 552 | 2014.11.29 | Male | 52 | ⅡA | T2 | N0 | 6.1 | lower esophagus | ulcerative type | middle | esophagogastrostomy over aortic arch(surgicalstapler) | >7 |
| 561 | 2014.12.22 | Female | 64 | ⅠB | T2 | N0 | 3.8 | middle esophagus | medullary type | high | esophagogastrostomy over aortic arch(surgicalstapler) | >7 |
| 566 | 2014.12.25 | Male | 75 | ⅠB | T1b | N0 | 3.7 | middle esophagus | mushroom type | high | Right intrathoracic anastomosis(surgicalstapler) | >7 |
| 567 | 2014.12.25 | Female | 66 | ⅡB | T3 | N0 | 3.7 | middle esophagus | ulcerative type | middle | esophagogastrostomy over aortic arch(surgicalstapler) | >7 |
| 573 | 2015.01.08 | Male | 62 | ⅡA | T3 | N0 | 6.3 | middle esophagus | ulcerative type | high | esophagogastrostomy over aortic arch(surgicalstapler) | >7 |
| 576 | 2015.01.15 | Male | 61 | ⅡA | T3 | N0 | 7.6 | lower esophagus | ulcerative type | middle-low | esophagogastrostomy over aortic arch(surgicalstapler) | >7 |
| 585 | 2015.2.5 | Male | 64 | ⅡB | T3 | N0 | 6.9 | middle esophagus | ulcerative type | middle-low | esophagogastrostomy over aortic arch(surgicalstapler) | >7 |
| 587 | 2015.2.12 | Female | 58 | ⅢB | T3 | N1 | 3 | middle esophagus | medullary type | middle-high | esophagogastrostomy over aortic arch(surgicalstapler) | >7 |
| 588 | 2015.2.13 | Female | 65 | ⅠB | T1b | N0 | 3 | middle esophagus | medullary type | middle | esophagogastrostomy over aortic arch(surgicalstapler) | >7 |
| 594 | 2015.3.9 | Male | 64 | ⅡA | T3 | N0 | 5.3 | lower esophagus | ulcerative type | high | esophagogastrostomy below aortic arch(surgicalstapler) | >7 |
| 603 | 2015.4.9 | Male | 60 | ⅡB | T3 | N0 | 5.2 | middle esophagus | ulcerative type | middle | esophagogastrostomy over aortic arch(surgicalstapler) | >7 |
| 604 | 2015.4.9 | Male | 66 | ⅡA | T3 | N0 | 7.1 | middle esophagus | medullary type | high | esophagogastrostomy over aortic arch(surgicalstapler) | >7 |
| 607 | 2015.4.14 | Male | 73 | ⅠB | T2 | N0 | 5.4 | middle esophagus | ulcerative type | high | manual anastomosis(neck) | >7 |
| 624 | 2015.5.19 | Female | 52 | ⅠB | T2 | N0 | 6.2 | middle esophagus | mushroom type | high | esophagogastrostomy over aortic arch(surgicalstapler) | >7 |
| 627 | 2015.5.25 | Male | 60 | ⅡA | T2 | N0 | 8.7 | middle esophagus | ulcerative type | middle | esophagogastrostomy over aortic arch(surgicalstapler) | 7 (75 mouths) |
| 631 | 2015.5.30 | Male | 73 | ⅠB | T2 | N0 | 7.3 | middle esophagus | ulcerative type | high | esophagogastrostomy below aortic arch(surgicalstapler) | >7 |
| 634 | 2015.6.15 | Male | 66 | ⅢB | T3 | N1 | 4.7 | middle esophagus | medullary type | middle | esophagogastrostomy over aortic arch(surgicalstapler) | >6 |
| 635 | 2015.6.15 | Female | 61 | ⅡB | T3 | N0 | 4.4 | middle esophagus | medullary type | middle | esophagogastrostomy over aortic arch(surgicalstapler) | >6 |
| 649 | 2015.7.24 | Female | 58 | ⅡA | T3 | N0 | 7.6 | lower esophagus | ulcerative type | high | esophagogastrostomy below aortic arch(surgicalstapler) | >6 |
| 665 | 2015.9.14 | Male | 63 | ⅡB | T3 | N0 | 2.9 | middle esophagus | ulcerative type | middle | esophagogastrostomy over aortic arch(surgicalstapler) | >6 |
| 669 | 2015.9.21 | Male | 63 | ⅡA | T3 | N0 | 4.2 | lower esophagus | ulcerative type | high | esophagogastrostomy over aortic arch(surgicalstapler) | >6 |
| 671 | 2015.9.23 | Male | 63 | ⅠB | T2 | N0 | 5 | lower esophagus | ulcerative type | high | esophagogastrostomy over aortic arch(surgicalstapler) | >6 |
| 677 | 2015.10.12 | Male | 66 | ⅡA | T3 | N0 | 6.5 | upper esophagus | ulcerative type | high | manual anastomosis(neck) | >6 |
| 679 | 2015.10.17 | Female | 66 | ⅠB | T1b | N0 | 4 | upper esophagus | ulcerative type | high | esophagogastrostomy over aortic arch(surgicalstapler) | >6 |
| 686 | 2015.11.09 | Female | 63 | ⅡA | T3 | N0 | 3.9 | middle esophagus | medullary type | high | esophagogastrostomy over aortic arch(surgicalstapler) | >6 |
| 712 | 2016.1.14 | Female | 67 | ⅡA | T3 | N0 | 4.5 | middle esophagus | ulcerative type | high | esophagogastrostomy over aortic arch(surgicalstapler) | >6 |
| 715 | 2016.1.28 | Female | 48 | ⅢB | T3 | N2 | 6.3 | middle esophagus | ulcerative type | high | esophagogastrostomy over aortic arch(surgicalstapler) | >6 |
| 720 | 2016.2.5 | Male | 58 | ⅡA | T2 | N0 | 2.8 | middle esophagus | ulcerative type | middle | esophagogastrostomy over aortic arch(surgicalstapler) | >6 |
| 728 | 2016.3.7 | Male | 62 | ⅡA | T2 | N0 | 8 | lower esophagus | medullary type | middle | esophagogastrostomy over aortic arch(surgicalstapler) | >6 |
| 760 | 2016.5.11 | Male | 65 | ⅡB | T3 | N0 | 5.2 | upper esophagus | ulcerative type | middle | esophagogastrostomy over aortic arch(surgicalstapler) | >6 |
| 788 | 2016.8.15 | Male | 69 | ⅡA | T3 | N0 | 5.3 | lower esophagus | ulcerative type | middle | esophagogastrostomy over aortic arch(surgicalstapler) | >5 |
| 789 | 2016.8.15 | Male | 73 | ⅡA | T2 | N0 | 3 | lower esophagus | ulcerative type | middle | esophagogastrostomy over aortic arch(surgicalstapler) | >5 |
| 793 | 2016.8.25 | Male | 66 | ⅡA | T3 | N0 | 5 | lower esophagus | medullary type | middle | esophagogastrostomy over aortic arch(surgicalstapler) | >5 |
| 801 | 2016.9.30 | Female | 64 | ⅢA | T2 | N1 | 5 | lower esophagus | ulcerative type | middle | esophagogastrostomy below aortic arch(surgicalstapler) | >5 |
| 822 | 2016.11.24 | Male | 73 | ⅡA | T2 | N0 | 5.8 | middle esophagus | ulcerative type | middle | esophagogastrostomy over aortic arch(surgicalstapler) | >5 |
| 858 | 2017.3.13 | Male | 48 | ⅡB | T3 | N0 | 5.1 | middle esophagus | ulcerative type | middle-high | esophagogastrostomy over aortic arch(surgicalstapler) | >5 |
| 877 | 2017.4.26 | Male | 73 | ⅠB | T1b | N0 | 4.7 | middle esophagus | protruded type | high | esophagogastrostomy over aortic arch(surgicalstapler) | >5 |
| 885 | 2017.5.19 | Male | 67 | ⅡA | T3 | N0 | 8.8 | lower esophagus | medullary type | middle | esophagogastrostomy over aortic arch(surgicalstapler) | >5 |
| 886 | 2017.5.19 | Male | 67 | ⅠB | T1b | N0 | 3 | middle esophagus | mushroom type | high | esophagogastrostomy over aortic arch(surgicalstapler) | >5 |
